# Making heads or tails of body inversion effects: Do heads matter?

**DOI:** 10.1371/journal.pone.0263902

**Published:** 2022-02-17

**Authors:** Emma L. Axelsson, Tharindi Buddhadasa, Laura Manca, Rachel A. Robbins

**Affiliations:** 1 School of Psychological Sciences, The University of Newcastle, Callaghan, Australia; 2 Research School of Psychology, The Australian National University, Canberra, Australia; University Hospitals Tubingen: Universitatsklinikum Tubingen, GERMANY

## Abstract

Observers are better at discriminating upright bodies than inverted bodies, and this body inversion effect (BIE) is reliable with whole figures (bodies with heads), but not with bodies presented without heads or the heads occluded suggesting that heads may be key to BIEs. Some studies present whole figures and bodies without heads between groups, and BIEs are not found for bodies without heads [1]. Other studies present whole figures and bodies without heads in the same blocks and BIEs are found with bodies without heads [2]. Does seeing the heads of whole figures induce BIEs in bodies without heads? Here, participants discriminated bodies with either whole figures and bodies without heads presented within blocks, or in separate blocks with bodies without heads presented first. We tested body identity *and* posture discrimination and measured participants’ gaze. BIEs were found with whole figures and bodies without heads in both identity and posture discrimination, and in both study designs. However, efficiency scores were better for the whole figures than the bodies without heads, but only when whole figures appeared in separate blocks. The magnitude of the BIE was overall stronger for whole figures compared to bodies without heads, but only in identity discrimination. BIE magnitudes were similar in the identity and posture tasks. Participants were better at identity discrimination, yet, there was greater looking at heads and less at bodies. During posture discrimination, greater looking at bodies and less at heads was associated with better performance. Faces might influence BIEs but are not essential. Configural representations of bodies without heads are sufficient for BIEs in posture and identity discrimination.

## The role of heads in body inversion effects in identity and posture discrimination

The finding that observers are faster and more accurate when discriminating pairs of upright compared to pairs of inverted faces is well established, and this inversion effect is stronger than that seen with other categories such as dogs or houses [3–6]. Inversion effects are also found with bodies and are similar in magnitude to the face inversion effect (FIE). Body inversion effects (BIEs) have been found in posture discrimination tasks where observers discriminate bodies with varying limb positions [7, 8]. BIEs are also seen with body identity discrimination where observers discriminate who people are based on bodies [2]. Faces and bodies are asymmetrical on the horizontal axis, and inversion affects the typical configuration of the features (eyes above the nose which is above the mouth; and arms at the top of torso, legs at the bottom), which in turn affects the discrimination of faces and bodies [2, 8]. There are regions of the brain that respond selectively to faces and bodies and as faces and bodies are typically seen in an upright orientation [9], inversion disrupts typical processing of bodies.

Body inversion effects (BIEs) have been found in multiple studies [see 10 for a review], but they are less reliable when bodies are presented without a head or the head occluded. In studies of body posture discrimination with 3D software-created images, Yovel et al. [1] found BIEs with whole figures, but not with bodies without heads (from here ‘headless bodies’). They did however, find BIEs with figures with missing arms or a missing leg, but with the head present. This suggests that the absence of BIEs with headless bodies was not due to a missing part in the figures, but that heads play an important role in the discrimination of bodies. The failure to find a headless BIE was replicated in follow-up studies [11, 12].

Subsequent findings with headless bodies have been inconsistent. Using the same stimuli as Yovel et al. [1], headless BIEs have been found in three other studies [13–15]. In a posture discrimination study with photographic images of real people [10], a headless BIE was also found with forward-facing headless figures, but not with headless bodies seen from behind (‘about-facing figures’). In studies of discrimination of the identities of bodies using photographic images, Minnebusch et al. [16] found no headless BIE based on accuracy and reaction time (RT) scores, and even a *reversed* headless BIE with efficiency scores such that participants more efficiently discriminated inverted compared to upright headless bodies. Contrastingly, Robbins and Coltheart [2], also using photographic images in a body identity discrimination task, found a headless BIE. However, the headless BIE was lower in magnitude than the whole figure BIE (with unfamiliarised stimuli). Ultimately, it appears that whole figure BIEs are consistently found, but headless BIEs are less consistently found across studies and tasks (posture and identity discrimination).

The above studies differ in several ways and they could explain the inconsistent findings. These include the type of stimuli (3D-software created or photographic), the sample sizes, the type of task (posture or identity discrimination), whether the study was a between or within groups design with participants seeing either whole figure or headless stimuli or both intermixed, and whether a sequential matching or match-to-sample method was used. Each of these are considered below.

When it comes to the **stimuli type**, headless BIEs are inconsistent with both 3D software-created [1, 13] and photographic images [2, 10, 16]. Therefore, the type of stimuli is unlikely to explain the inconsistent headless BIEs. In the studies here, photographic images were used as the stimuli came from previous studies [2, 10].

The **sample sizes** differed across studies. Weaker BIEs were found for headless compared to whole figures in posture [13] and identity discrimination tasks [2]. If there is an inversion effect with headless images, but it is a weak effect, then sufficient power is needed to observe an effect. In posture discrimination tasks, Axelsson et al. [10] largely found medium effect sizes with whole figures and small effect sizes with headless figures. In posture discrimination studies where headless BIEs were not found, the samples sizes were smaller [1: n = 12 per condition, 12: n = 14 per condition]; but studies finding headless BIEs had slightly larger sample sizes [10: n = 28, 13: n = 16, 14: n = 70+]. For body identity discrimination studies with inconsistent results, the sample sizes were also smaller [16: n = 17]; but studies finding headless BIEs had larger sample sizes [2: n = 24 with familiarised bodies & n = 40 with unfamiliar bodies]. In the current studies, we used *n* = 28 in each as based on a power analysis and a previous study [10].

Does the **type of body discrimination** matter? Headless BIEs in *posture* discrimination tasks have been inconsistent across studies even with the same stimuli [1, 13]. There are only two known studies investigating headless BIEs in *identity* discrimination, but they used different stimuli [2, 16]. In the images of Minnebusch et al. [16], people in the images wore a variety of clothing. Participants might have relied more on clothing cues than identity and this could explain the reversed BIE and better performance with the inverted images. Inverted images are associated with more piecemeal, featural as opposed to configural processing [8]. In Robbins and Coltheart’s study [2], people wore only black clothing and participants were likely less able to rely on clothing and more on identity. Therefore, there are inconsistencies across both types of tasks. One of the aims of the current study is to test the headless BIE in both body identity and posture discrimination, but to limit variability in clothing between body pairs.

Another difference is whether the studies had a **within or between groups design**. Many had a within groups design with whole and headless bodies presented within the same blocks [2, 10, 16]. Yovel et al. [1] used a between groups design with participants seeing either whole or headless bodies. As Yovel et al. [1] found a BIE with whole, but not headless figures, they argued that implied facial information from the heads likely drives configural processing of upright whole figures; and in turn an inversion effect as inversion disrupts configural processing. However, as a between groups design was used, the difference in results with whole and headless bodies could have been due to individual differences in their participants.

Further evidence suggesting that facial information drives a BIE comes from Brandman and Yovel [11] who found that body selective areas (extrastriate body area (EBA) and fusiform body area (FBA)) were sensitive to upright and inverted, whole and headless bodies. Contrastingly, face selective parts of the brain (fusiform face area (FFA) and the occipital face area (OFA)) were sensitive to only upright whole figures even though the facial features were removed. Therefore, it was argued that the greater involvement of face selective areas drives configural processing in discrimination of upright whole figures. Brandman and Yovel [12] also found that with brief presentations (27 milliseconds) of whole figures (faceless) and headless figures, participants rated themselves as more certain they had seen a face with the whole figures compared to headless bodies. They argued that the faceless whole figures provide a form of contextual priming of configural processing associated with faces. If faces, seen or imagined, are critical to a BIE, then in within group designs, implied faces from the heads of whole figures might carry over to the headless trials and contribute to a headless BIE. Repeatedly seeing whole figures might induce BIEs with headless bodies due to repetition priming [see also 10].

However, if repetition priming is the explanation for headless BIEs, then we should reliably see headless BIEs in within groups designs where participants see whole and headless figures intermixed in the same blocks. For identity discrimination, Robbins and Coltheart [2] presented whole and headless bodies in the same blocks and found a headless BIE. However, Minnebusch et al. [16], using a similar design, did not find a headless BIE in identity discrimination. In posture discrimination tasks, Axelsson et al. [10] presented whole and headless bodies intermixed in the same blocks and had mixed results. They found a headless BIE with forward-facing postures, but only with *d* prime and not with efficiency scores, and BIEs were not found with about-facing headless images (images seen from behind). The authors concluded instead that BIEs are more likely to be found with more typical presentations of bodies (i.e., upright body with head and forward facing). Arizpe et al. [13] used a within groups design, but participants saw whole and headless postures on separate days, which should reduce any priming, yet they found a headless BIE. Therefore, repetition priming from the heads of whole figures might not induce headless BIEs. To further test the effects of repetition priming, in the current study, two different groups of participants were tested: one group saw whole and headless bodies intermixed within the same blocks and another groups saw headless and whole figures in separate blocks with the headless bodies always appearing first to limit repetition priming of facial information from the whole figures. Participants discriminated pairs of whole and pairs of headless bodies in identity and posture tasks to allow for a comparison within participants.

The **method** of the studies also differed. Most used sequential matching where participants indicate whether a test image is the same or different to a previously presented image [1, 8, 10, 13, 14, 16]. One downside of sequential matching is that participants need to remember which of two response buttons correspond to ‘same’ or ‘different’ options and this could involve greater error or cognitive load. This could be an issue if the headless BIE is a weak effect. With a match-to-sample method, participants decide which of *two* test images match the body shown previously, and this involves pressing a button that corresponds to the same side as their chosen image (e.g., button on left side of keyboard corresponds to the left image). Robbins and Coltheart [2] found a headless BIE in their identity discrimination task using a match-to-sample method. Studies with inconsistent headless BIEs [10, 16] used sequential matching. The method might not explain the differing findings, but the match-to-sample method will be used here to attempt to reduce button-choice-related error.

**Here**, using a within groups design, participants discriminated body postures with the same stimuli as used in Axelsson et al. [10 forward-facing images]. They also discriminated identities with the same stimuli as used in Robbins and Coltheart [2]. One group of participants saw whole and headless figures intermixed within blocks (intermixed group) and another group saw headless stimuli separately to and before seeing whole figures (blocked group) to limit repetition priming of facial information.

**Fig 1:**
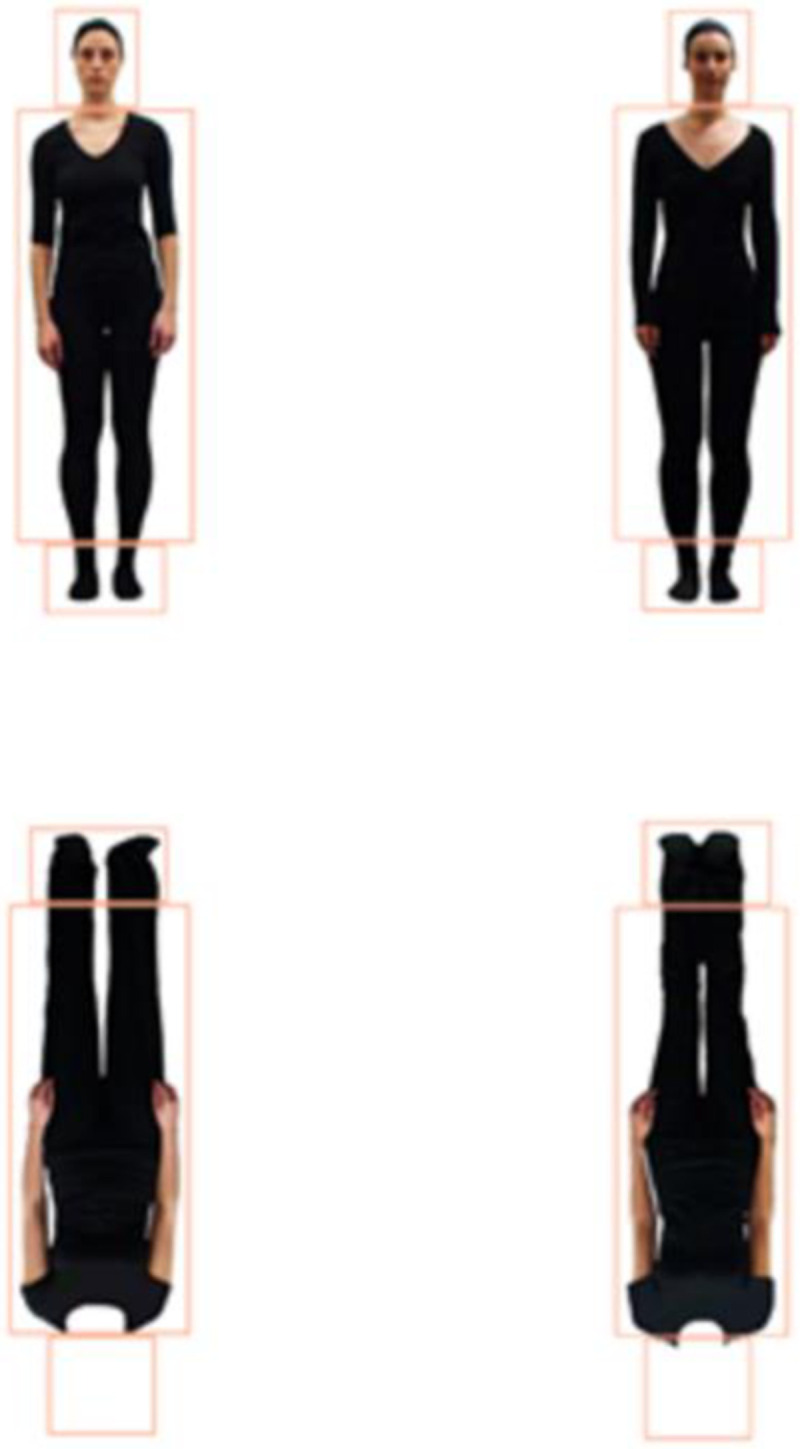
Identity task whole figure upright (WFU) pair and headless inverted (HLI) pair along with interest areas (IAs) surrounding the heads, bodies, and feet.

**Fig 2:**
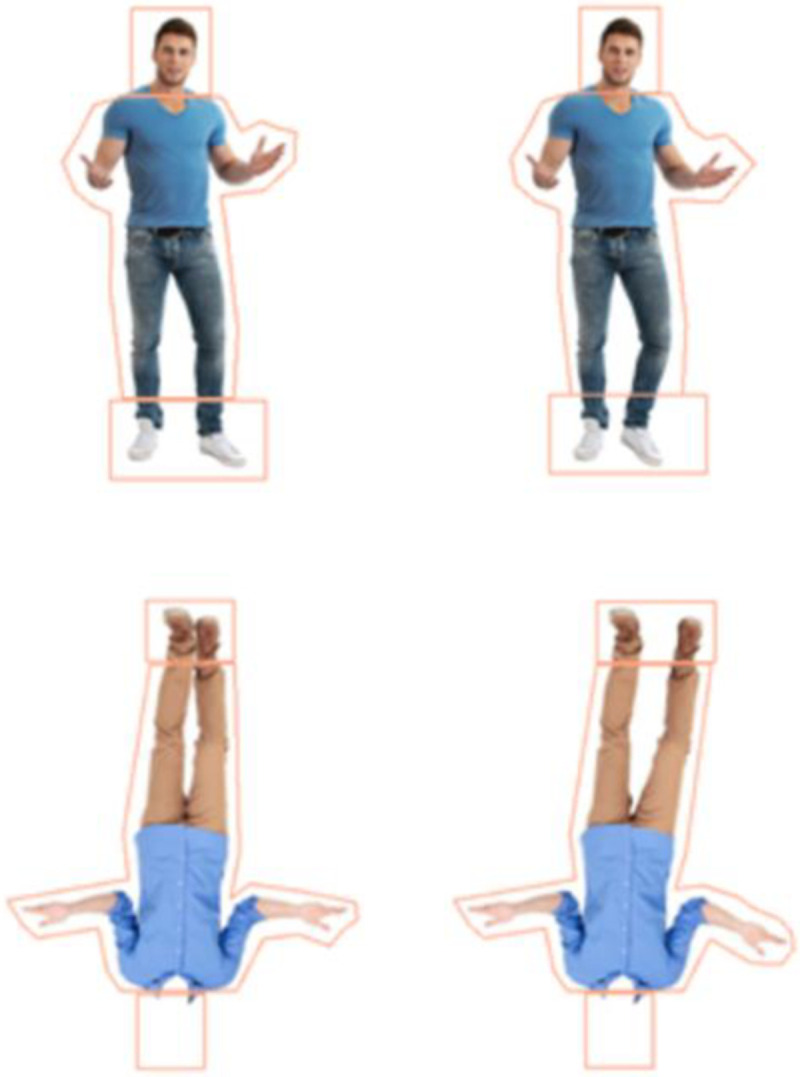
Posture task whole figure upright (WFU) pair and headless inverted (HLI) pair along with interest areas (IAs) surrounding the heads, bodies, and feet.

Given the consistent inversion effects with whole figures [1, 2, 10, 13, 16], it was expected that BIEs would be found in both studies in body identity and posture discrimination. However, for the headless images the predictions were less certain. For identity discrimination, as the stimuli were the same as those used in Robbins and Coltheart [2], headless BIEs were expected for the intermixed group with the whole figure and headless figures presented within blocks. However, if Robbins and Coltheart’s [2] findings were due to contextual or repetitive priming, it was expected that a BIE might not be found when headless images were presented on their own for the blocked group. For posture discrimination, headless BIEs have been inconsistent [1, 10, 13], but as a headless BIE was found with the same forward facing headless BIEs in Axelsson et al. [10] it was expected to be found here. The predictions for a headless BIE in the blocked study where headless stimuli appear alone were tentative. We also expected that any headless BIEs would be weaker than the whole figure BIEs as seen previously [2].

We measured participants’ gaze. Arizpe et al. [13] found that participants tended to focus more on the upper torso and head of upright whole figures and upper torso of upright headless bodies. For inverted figures there was a focus on the lower torso. Instructing participants to focus on the heads or upper torso was also associated with better performance, but only for the whole figures, not the headless. Axelsson et al. [10] also found a greater focus on the heads of upright compared to inverted postures, and a greater focus on the bodies of headless stimuli than the bodies of whole figures. This suggests that focussing on bodies does not necessarily correspond with better body discrimination as effect sizes were higher in the whole figure conditions. They did however, find better performance with inverted images when participants focussed more on the bodies and less on the heads of whole figures or feet of headless figures. It was therefore predicted here that when the images were inverted a greater focus on bodies would be associated with better performance, but with upright images a great focus on the upright regions would be associated with better performance.

## Method

### Participants

A power analysis, based on Yovel et al.’s [1] effect size of η_*p*_^2^ = .33, indicated that a sample size of 28 was required per group. This was the sample size used in Axelsson et al. [10] who presented the same body posture images and is similar to the sample size in Robbins and Coltheart [2] where the body identity images were sourced (familiarised bodies, *n* = 24; unfamiliarised bodies, *n* = 40). In the intermixed group, the final sample of 28 had a mean age of 20.91 years (*SD* = 1.65, range = 18.96–26.03, 22 female; 17 white/European descent & 11 Asian). Eye tracking data was missing from one participant, but behavioural data was collected. A further two were tested, but had difficulties completing the task. In the blocked group, the final sample of 28 adults had a mean age of 20.87 years (*SD* = 1.89, range = 18.05–24.88, 17 female; 19 Caucasian & 9 Asian). A further two were tested, but the data for one was removed due to eye fatigue and one due to difficulties completing the task. All participants reported normal or corrected-to-normal vision. Participants were recruited via the research participation website (SONA) at The Australian National University (ANU) for course credit or through a flyer posted on social media. Social media recruits received AUD 15 for participating. The studies were conducted in compliance with ethical standards and was approved by the university’s Human Research Ethics Committee (Protocol number 2015/183). Written consent was provided by the participants.

### Apparatus

#### Eye tracker

An EyeLink 1000 eye tracker (SR research, http://www.sr-research.com) recorded participants’ eye movements in a darkened room by recording the infrared reflections from the cornea and pupil. The ‘Desktop Mount’ set-up was used with a 35 mm camera lens, which tracked one of the eyes at a sampling frequency of 1000 Hz, and an average spatial accuracy of 0.15°. The camera was directly below and in front of a 24-inch Dell computer monitor with a screen resolution of 1920 × 1080 pixels. A chin rest was used to stabilise the participants’ heads 70 cm from the camera and 90cm from the display screen.

### Stimuli

For both the identity and posture discrimination tasks, there were four body type conditions (see Figs 1 and 2): whole figure upright (WFU), whole figure inverted (WFI), headless upright (HLU), and headless inverted (HLI). In each task (identity & posture discrimination), there were 12 pairs of figures and each was presented in the four body type conditions resulting in 48 pairs of figures for each task. Faces were shown in the whole figures in both tasks as they were shown in the original studies [2, 10], and this is the typical appearance of people. The headless images were the main interest here. To create the headless stimuli, the heads of the whole figure stimuli were erased from the top of the upper garment of clothing using Adobe Photoshop CS6 (see Figs 1 and 2). Inverted stimuli were created by rotating the images 180°.

#### Identity discrimination task

These stimuli were from Robbins and Coltheart [2]. There were 12 pairs of identities (24 Caucasian females). For each identity there were two different photographs (48 images). As a match-to-sample method was used, a different photograph of the initial identity was shown at test, paired with a new identity, and participants were required to choose which identity matched the initial one. The use of two different photographs for each identity was to enhance the likelihood that participants would engage in identity matching rather than matching of photographs [see 17]. The figures stood with their arms by their sides, and wore plain, black shirts, black trousers, and black fitted hats (see Fig 1). All jewellery was removed. As in Robbins and Coltheart [2], the figures in each pair wore similar clothing (e.g., short or long sleeves) and had similar body shapes to enhance the likelihood that identity discrimination would be based on identity and less on clothing and body shape. The average size of the whole figures was 4.96°×12.50° visual angle at 900mm distance and the headless figures were 4.96°×10.82°.

#### Posture discrimination task

Twelve pairs of stimuli were sourced from Axelsson et al. [10]. These were photographs of forward-facing adult Caucasian male human figures, sourced from Shutterstock.com. All figures had short, dark brown hair and wore similar clothing (plain, blue shirts and trousers) to reduce attention to clothing or hair. They all had similar body shapes. The figures in each pair were the same identity with the same clothing and differed only in posture (see Fig 2). Using Adobe Photoshop (CS6), the postures were altered in 2D space by rotating or shifting the limbs of the figures up or down. Four pairs differed in both an arm and leg position, four pairs in only a leg position, and four pairs in only an arm position. The angle of the limb positions were changed in Photoshop. For the pairs that differed in the arm and leg positions, the angles were altered by 10–15˚. For the pairs that differed in only one limb (arm or leg), the angles were altered by 20–30˚. All poses are biologically possible as tested by one of the authors (ELA). Head positions in the whole figure conditions were identical within a pair. The average size of the whole figures was 8.44°×12.84° visual angle at 900mm distance and the headless figures were 8.44°×11.36°.

Ideally, the same gender would have been presented in both tasks, but the original identity and posture discrimination studies [2, 10] were created independently and used different genders. The same stimuli were used here to aid in comparability of the results. Gender was also expected to have less of an effect on performance, and the task type, orientation, and whether the bodies were headless or not, were expected to be more relevant to any inversion effects. Nonetheless, due to the differing genders in the images, we ran extra analyses to see if there were any effects on the genders of the participants to see if they responded differently to the images. These analyses with participant gender are presented in the Supplementary Materials.

### Procedure and design

**Fig 3:**
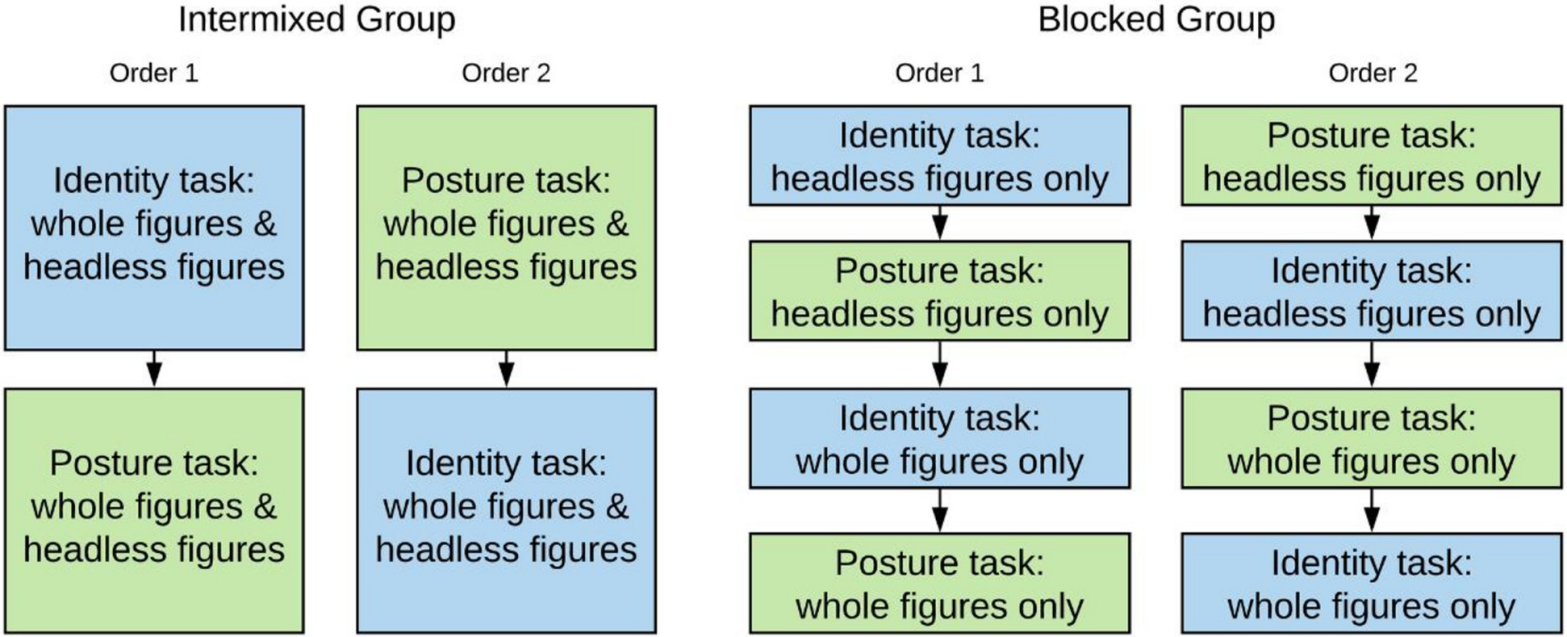
Design difference between the intermixed and blocked groups. The presentation order of identity and posture tasks was counterbalanced across participants.

A 2 (task type: identity, posture discrimination) × 2 (orientation: upright, inverted) × 2 (body type: whole figure, headless) × 2 (group: body type intermixed, body type blocked) mixed model design was used. The within groups variables were task type, orientation, and body type, but the identity and posture discrimination tasks were completed in separate blocks, and the presentation order of the two tasks was counterbalanced across participants (see Fig 3). The between groups factor was whether participants saw whole and headless figures intermixed in blocks (intermixed group) or in separate blocks (blocked group). For the blocked group, headless and whole figure stimuli appeared in separate blocks and the headless figure blocks appeared before the whole figures to prevent repetition priming of head information in the headless conditions (see Fig 3). For both groups, upright and inverted figures were presented within each block. All participants saw a certain pair of images in a certain body type condition (WFU, WFI, HLU, HLI) an equal number of times (e.g., Pair 1 appeared twice in the WFU condition). For the intermixed group, there were four versions of the task which counterbalanced the order of presentation of the body type conditions (WFU, WFI, HLU, HLI). For the blocked group, the presentation of orientation was randomised. For both groups, each consecutively presented pair of figures differed to ensure that participants were discriminating between pairs based on identity or posture and not comparing to a previous presentation. A given pair therefore, appeared once every eight trials to ensure the presentation of individual pairs was spread out.

The experimenter provided instructions before commencement of each block and a set of written instructions were also presented on the display monitor. Calibration and validation were performed using the standard EyeLink 1000 nine-point display and was repeated if necessary until fixations were less than 1° visual angle from the calibration points. A drift correction preceded every trial to ensure the participants remained calibrated and centrally fixated. Using a match-to-sample method, the initial image appeared for 250 ms, followed by a 1000 ms inter-stimulus interval (a blank off-white screen), and then a pair of test figures, one that matched the initial image and the other that did not. In the identity task, the matched image was a different photograph of the same identity as the initial image, and the other test image was a different identity. In the posture task, the matched image was the same image as the initial image and the other test image had a slightly different posture. A maximum of 5000 ms was given for participants to indicate which of the two test images matched the initial image. Participants were instructed to respond as quickly, yet as accurately as possible. If the participants did not respond within 5000 ms, the trial was listed as having had no response and we excluded these from analyses. A standard (ANSI) QWERTY keyboard (for the English language) was used and participants pressed the ‘z’ key on a keyboard if they thought the matching image was on the left and the ‘/’ key if they thought it was on the right. The ‘z’ and ‘/’ keys are on the second row of the keyboard and are sufficiently distanced from each other and allow participants to rest their hands comfortably on the table shoulder-width apart. The response keys were marked with Velcro (smooth for ‘z’ and rough for ‘/’) as tactile reminders to prevent participants from accidentally pressing the neighbouring keys given they were in a darkened room and head movements were restricted due to the chinrest. Each identity or posture in a pair was presented as the initial image an equal number of times. Each identity or posture in a pair always appeared on the left or right of the screen, but the left/right position of the image matching the initial image was randomised across trials. There was an equal number of trials where the matched image appeared on the left or right side. Participants were given a short break of 2–5 minutes before commencement of the next block. For the intermixed group, in each task, participants completed eight practice trials followed by 96 experimental trials. In each task, there were 48 pairs of stimuli and each pair was presented twice, once in the first half, once in the second half with the side position of the matching images switched for the second presentation. The practice stimuli did not reappear in the experimental trials. The procedure for both tasks was identical and each task ran for approximately 10 minutes. For the blocked group, there four practice trials prior to each task and 48 experimental trials in each task (24 pairs of stimuli presented twice across the block).

## Behavioural results

**Fig 4:**
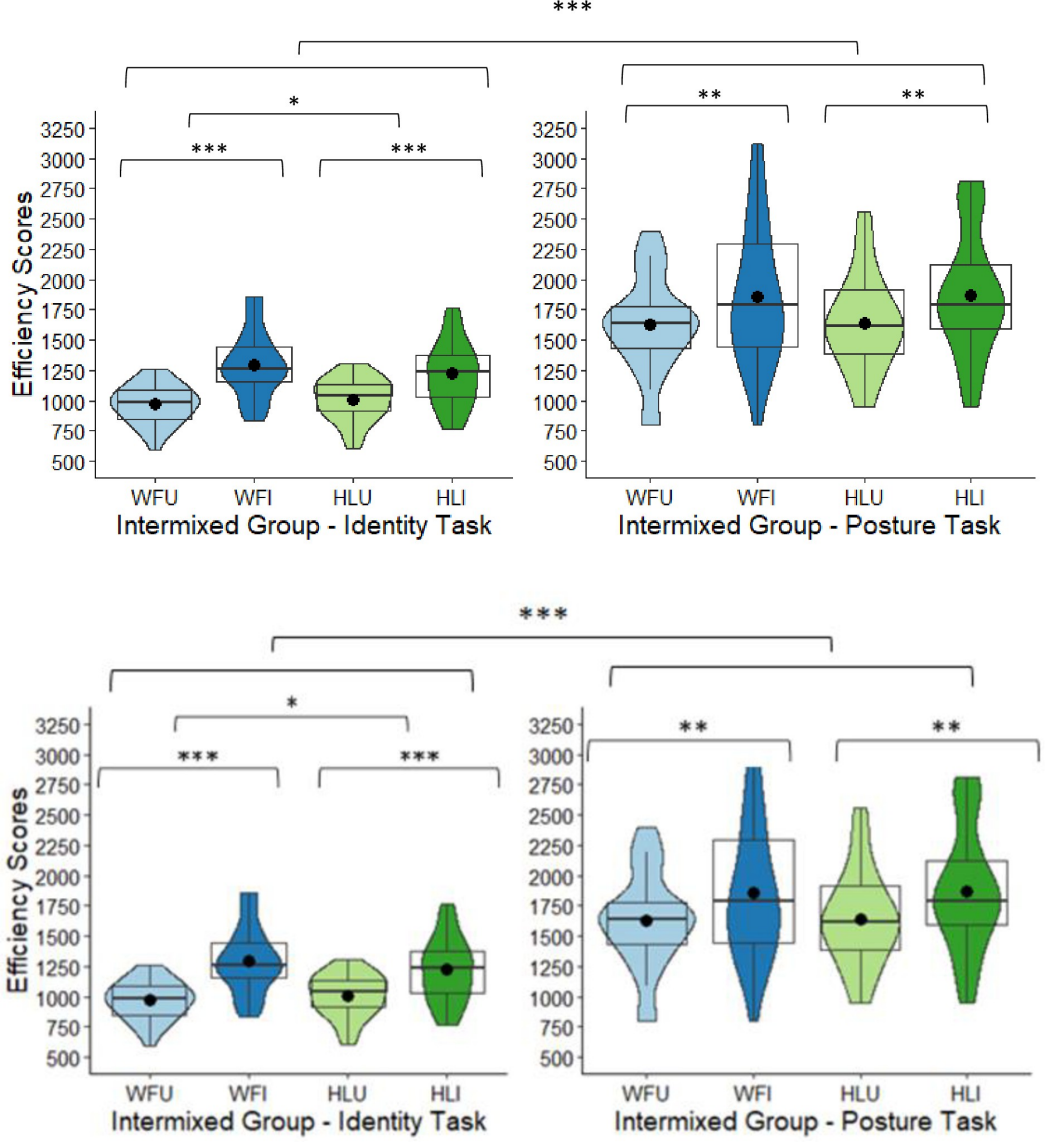
Intermixed group’s box and violin plots of efficiency scores for the four conditions: Whole figure upright (WFU), whole figure inverted (WFI), headless upright (HLU), headless inverted (HLI) in the identity task and posture task; dots denote means; *** = p < .001; ** = p < .01; * = p < .05.

Data was extracted using Data Viewer 3.1.1 (SR Research) and analysed using IBM SPSS 25. The graphs were made in R Studio 1.1.456. Inverse efficiency scores were analysed to account for speed/accuracy trade-offs and were calculated by dividing the mean RTs in correct trials by the proportion of correct responses for each participant in each condition [16, 18]. This is because participants were asked to respond as quickly and accurately as possible [but see 18–21 for discussions on this]. To directly test for inversion effects, planned paired *t*-tests were performed comparing performance between the upright and inverted images for each body type and each task type. In the intermixed group, the identity task had one outlier (*z*-score >2.5 *SD*) in the WFU condition and one in the HLU condition; and in the posture task, there was one in the HLI condition. In the blocked group, the identity task had one outlier in the WFU and one in the WFI condition. All outliers were replaced with the condition means. A 2 × 2 × 2 × 2 mixed model ANOVA was performed to compare efficiency scores across the two groups (body types intermixed, body types blocked), between the two tasks (identity, posture), between the two body types (whole figure, headless), and the two orientations (upright, inverted). The main effect of group was non-significant (see Table 1 and Fig 4). The main effect of task was significant. Scores were overall more efficient in the identity (*M* = 1127.78, *SD* = 258.06) than in the posture task (*M* = 1665.93, *SD* = 473.90). The main effect of body type was significant. Scores were overall more efficient in the whole figure (*M* = 1347.12, *SD* = 358.55) than in the headless condition (*M* = 1446.59, *SD* = 373.41). The main effect of orientation was significant as scores were overall more efficient in the upright (*M* = 1274.06, *SD* = 311.96) than in the inverted conditions (*M* = 1519.65, *SD* = 420.00). There were a number of significant interactions (see Table 1).

**Table 1:** Behavioural results: Main effects and interactions.

Effect	
Group	*F*(1,54) = 1.03, *p* = .315, η_*p*_^2^ = .02
Task	***F*(1,54) = 183.21, *p* < .001, η**_***p***_^**2**^ **= .77**
Body type	***F*(1,54) = 22.42, *p* = < .001, η**_***p***_^**2**^ **= .29**
Orientation	***F*(1,54) = 94.56, *p* = < .001, η**_***p***_^**2**^ **= .64**
Group × task	***F*(1,54) = 4.93, *p* = .031, η**_***p***_^**2**^ **= .08**
Group × body type	***F*(1,54) = 23.97, *p* = < .001, η**_***p***_^**2**^ **= .31**
Group × orientation	*F*(1,54) = 0.04, *p* = .839, η_*p*_^2^ = .00
Task × body type	*F*(1,54) = 2.73, *p* = .105, η_*p*_^2^ = .05
Task × orientation	*F*(1,54) = 0.02, *p* = .891, η_*p*_^2^ = .00
Body type × orientation	***F*(1,54) = 5.87, *p* = .019, η**_***p***_^**2**^ **= .10**
Group × task × body type	*F*(1,54) = 1.09, *p* = .301, η_*p*_^2^ = .02
Group × task × orientation	*F*(1,54) = 0.57, *p* = .452, η_*p*_^2^ = .01
Group × body type × orientation	*F*(1,54) = 0.21, *p* = .646, η_*p*_^2^ = .00
Task × body type × orientation	***F*(1,54) = 6.03, *p* = .017, η**_***p***_^**2**^ **= .10**
Group × task × body type × orientation	*F*(1,54) = 0.13, *p* = .723, η_*p*_^2^ = .00

The interaction between group and task was significant. The tasks were compared in each group to see whether participants performed differently across tasks depending on whether the whole and headless stimuli were blocked or intermixed. In the intermixed group, scores were significantly more efficient in the identity (*M* = 1123.84, *SD* = 189.56) than the posture task (*M* = 1750.22, *SD* = 417.38), *t*(27) = -9.82, *p* < .001, *d* = -1.86. In the blocked group, efficiency scores were also significantly more efficient in the identity (*M* = 1131.73, *SD* = 248.12) than in the posture task (*M* = 1581.64, *SD* = 410.14), *t*(54) = -9.48, *p* < .001, *d* = -1.79. The interaction is likely due to the larger difference in the intermixed group. The interaction between group and body type was also significant. The body types were compared in each group to see if intermixing or blocking headless and whole figures had an effect on any differences in efficiency between the whole and headless figure conditions. In the intermixed group, where whole and headless figures appeared in the same blocks, the difference in overall efficiency scores between the whole figure (*M* = 1438.72, *SD* = 347.03) and headless conditions (*M* = 1435.84, *SD* = 337.23) was non-significant, *t*(27) = 0.17, *p* = .865, *d* = 0.01. However, in the blocked group, where headless figures appeared first and separately to whole figures, efficiency scores were significantly more efficient in the whole figure (*M* = 1255.52, *SD* = 349.36) than in the headless condition (*M* = 1457.84, *SD* = 403.67), *t*(27) = -5.45, *p* < .001, *d* = 0.56. Therefore, presenting whole and headless figures separately led to an overall difference in performance in body types. The interactions between body type and orientation, and between task, body type, and orientation were also significant. These were explored below with planned contrasts comparing upright and inverted bodies in each task and for each body type.

### Tests of inversion: Intermixed group

In the identity task, for the whole figure images, scores were significantly more efficient in the upright (WFU: *M* = 969.34, *SD* = 162.12) than in the inverted condition (WFI: *M* = 1296.91, *SD* = 270.51), *t*(27) = 7.78, *p* < .001, Cohen’s *d* = 1.47. For the headless identity images, the difference between the upright (HLU: *M* = 1008.19, *SD* = 177.10) and inverted condition (HLI: *M* = 1220.91, *SD* = 269.82) was also significant, *t*(27) = 5.33, *p* < .001, *d* = 1.01. The magnitude of the BIE (difference between upright and inverted figures) was also significantly larger in the whole figure (*M* = -285.89, *SD* = 203.83) than in the headless condition (*M* = -178.52, *SD* = 199.06), with a correction applied, *t*(27) = 2.53, *p* = .018, Bonferroni-corrected (α × 2) = .036, *d* = 0.48 (see Fig 4). Therefore, for the identity task, there was a BIE in the whole figure and headless conditions, but the BIE was larger in the whole figure than in the headless condition.

In the posture task, for the whole figure images, scores were significantly more efficient in the upright (WFU: *M* = 1626.70, *SD* = 390.46) than in the inverted condition (WFI: *M* = 1861.93, *SD* = 565.03), *t*(27) = 3.35, *p* = .002, Cohen’s *d* = 0.60. For the headless posture images, the difference between the upright (HLU: *M* = 1642.44, *SD* = 398.10) and inverted condition (HLI: *M* = 1869.82, *SD* = 503.91) was also significant, *t*(27) = 3.20, *p* = .004, *d* = 0.60. However, the magnitude of the difference between the upright and inverted images between the whole figure (*M* = -235.23, *SD* = 371.08) and the headless conditions (*M* = -227.38, *SD* = 376.25) was non-significant, *t*(27) = 0.10, *p* = .919, corrected (α × 2) = 1.00, *d* = 0.02 (see Fig 4). Therefore, for the posture task, there was a BIE in the whole figure and headless conditions, but the difference in magnitude of the BIE between the whole figure and headless conditions was non-significant.

Given the significant task by body type by orientation interaction, the magnitude of the inversion effects across task types was also compared. The magnitude of the inversion effects across the identity and posture tasks did not differ significantly for the whole figures, *t*(27) = 0.80, *p* = .428, corrected (α × 4) = 1.00, *d* = 0.15, or for the headless figures, *t*(27) = -0.64, *p* = .526, corrected (α × 4) = 1.00, *d* = 0.12 (see Fig 4).

### Tests of inversion: Blocked group

**Fig 5:**
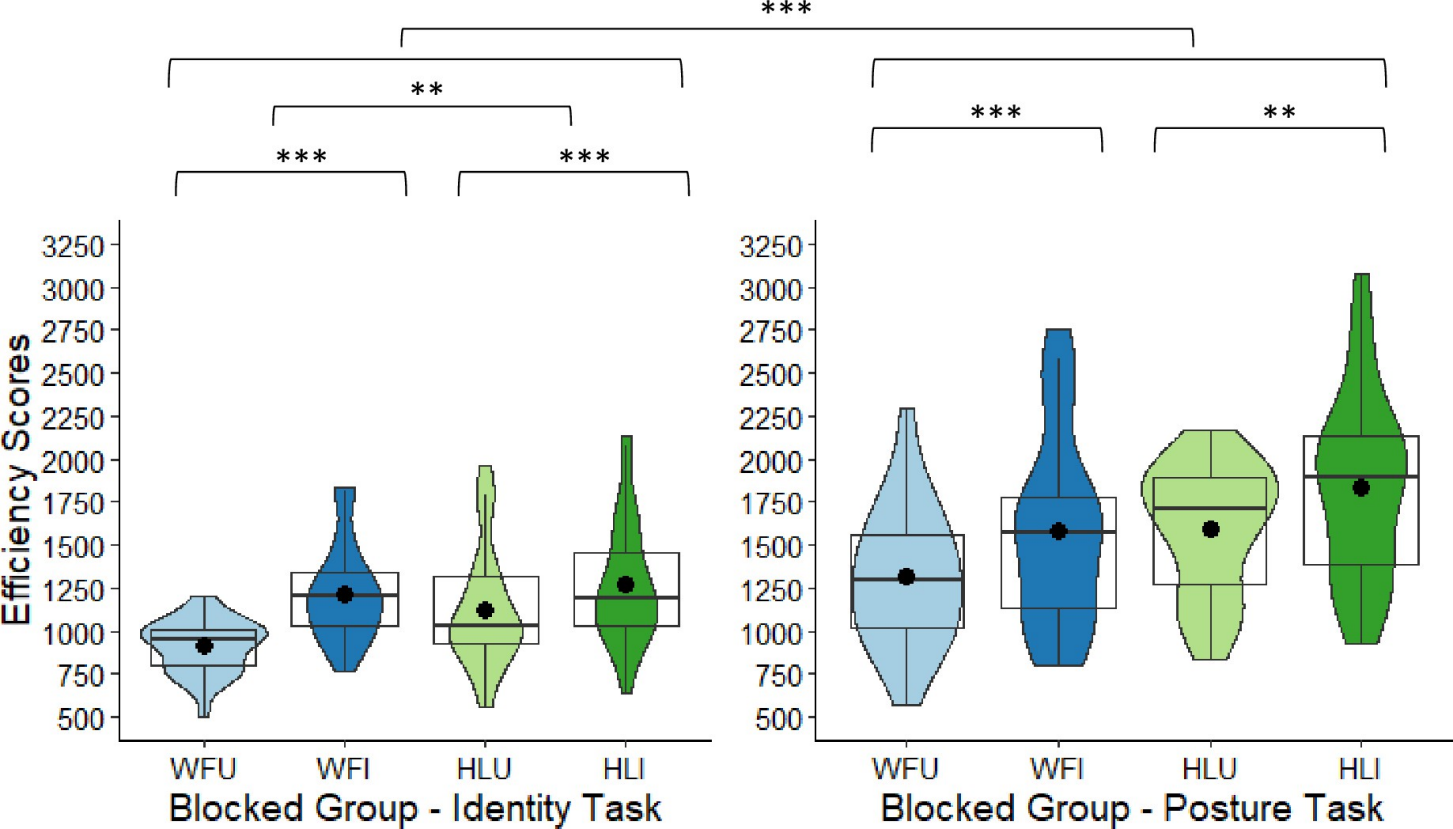
Blocked group’s box and violin plots of efficiency scores for the four conditions: Whole figure upright (WFU), whole figure inverted (WFI), headless upright (HLU), headless inverted (HLI) in the identity task and posture task; dots denote means; *** = *p* < .001; ** = *p* < .01.

In the identity task, for the whole figures, scores were significantly more efficient in the upright (WFU: *M* = 910.30, *SD* = 157.28) than in the inverted condition (WFI: *M* = 1218.26, *SD* = 281.10), *t*(27) = 7.40, *p* < .001, Cohen’s *d* = 1.40. For the headless identity images, the difference between the upright (HLU: *M* = 1126.14, *SD* = 347.37) and inverted condition (HLI: *M* = 1272.21, *SD* = 358.44) was also significant, *t*(27) = 3.40, *p* = .002, *d* = 0.64. Therefore, for the identity task, there was a BIE in the whole figure and headless conditions, and the magnitude of the BIE was significantly larger in the whole figure (*M* = -307.96, *SD* = 220.30) than in the headless condition (*M* = -122.13, *SD* = 186.65), with a correction applied, *t*(27) = 3.95, *p* = .001, corrected (α × 2) = .002, *d* = 0.75 (see Fig 5). This was the same pattern found when whole and headless bodies were intermixed.

In the posture task, for the whole figure images, scores were significantly more efficient in the upright (WFU: *M* = 1315.16, *SD* = 404.14) than in the inverted condition (WFI: *M* = 1578.36, *SD* = 554.90), *t*(27) = 5.18, *p* < .001, Cohen’s *d* = 0.98. For the headless posture images, the difference between the upright (HLU: *M* = 1594.24, *SD* = 373.52) and inverted condition (HLI: *M* = 1838.78, *SD* = 535.37) was also significant, *t*(27) = 3.56, *p* = .001, *d* = 0.67. Therefore, for the posture task, there were BIEs in both the whole figure and headless conditions, but the magnitude of the difference between the upright and inverted images (i.e., the BIE) between the whole figure (*M* = -263.20, *SD* = 268.68) and the headless conditions (*M* = -244.54, *SD* = 363.77) was non-significant, *t*(27) = 0.32, *p* = .752, corrected (α × 2) = 1.00, *d* = 0.06 (see Fig 5). Again, this was the same pattern found when whole and headless bodies were intermixed.

Given the significant task by body type by orientation interaction, the magnitude of the inversion effects across task types was also compared. The magnitude of the inversion effects across the identity and posture tasks did not differ significantly for the whole figures, *t*(27) = 0.80, *p* = .429, corrected (α × 4) = 1.00, *d* = 0.15, or for the headless figures, *t*(27) = 1.54, *p* = .136, corrected (α × 4) = 0.544, *d* = 0.25 (see Fig 5).

Note, as the genders in the images of the identity (female) and posture task (male) differed, we ran extra analyses to check whether participant gender played a role. These analyses were performed using linear mixed effects models due to the unequal numbers of female and male participants. The main effect of participant gender was non-significant, but there was a significant group × gender and group × task × gender interaction (see Table A1 in S1 File). Follow-up simple effects comparisons were performed comparing efficiency scores between female and male participants in the identity and posture tasks in the intermixed and blocked groups. Male participants in the blocked group were significantly more efficient than female in the posture task where male images are shown (see Tables A2 and A3 in S1 File).

**To summarise,** in the intermixed and blocked groups, there was overall better performance in the identity task, but BIEs were found in both the identity and posture tasks, and for the whole figure and headless images. There was also a significant task by body type by orientation interaction. This is explained by the larger BIE magnitude for the whole figures than in the headless figures in the identity task, but in the posture task the BIE magnitude did not differ between whole and headless figures. One key question was whether a headless BIE is explained by the headless stimuli appearing intermixed in blocks with whole figures and whether seeing the faces of whole figures might induce a BIE in the headless trials. However, in the blocked group, where headless and whole figures appeared separately and the headless images appeared first, there was the same pattern of inversion effects seen in the intermixed group. Therefore, there were BIEs when headless images were presented in blocks first without any whole figures, suggesting it is unlikely that any headless BIEs in the intermixed group were induced by being presented in blocks with whole figures with heads. There was however one difference between the studies. There was a significant interaction between body type and group. In the blocked group, scores were significantly more efficient for the whole compared to the headless figures. This difference was non-significant in the intermixed group. Perhaps presenting headless images with whole figures in the intermixed group led to similar efficiency with the headless and whole figures. Finally, for both groups, the differences in magnitude in BIE between the identity and posture tasks were non-significant for both the whole and headless conditions. Therefore, despite better overall performance in the identity task, the BIEs were similar in both identity and posture discrimination. This also suggests the differing faces in the identity pairs and identical faces in the posture pairs did not lead to differences in BIEs across the two task types. Regardless, the key interest here was in the headless bodies. With the participant gender analyses, there was an indication that male participants were more efficient than female for the posture task, which involved male images. This was only for the blocked group where whole and headless images appeared separately.

## Eye tracking data

Using Data Viewer software version 3.2.48 (SR Research), polygonal interest areas (IAs) were superimposed on the heads, bodies, and feet of all the figures. The IAs around the heads covered the head and neck, the IAs around the bodies extended from the ankles to the top of the torso, and the feet IAs were around the feet (see Figs 1 and 2). Feet were of interest here because for the inverted figures the feet appear where the head of the upright whole figures are seen. As participants demonstrate an upper bias [10, 13], would this apply to feet when the figures are inverted? This also allows for comparison to Axelsson et al. [10] who found that greater looking to the feet of inverted figures was associated with poorer performance. Dwell time (DT) is the sum of the durations of all the fixations within an IA. DTs to the IAs of the test images were averaged across all trials in each condition. DT proportions to each IA (head, body, feet) were calculated for each participant by dividing the DT to each IA by the DTs to all three IAs (e.g., DT to head/(head + body + feet)). As heads, bodies, and feet are presented simultaneously, to avoid violating the assumption of independence, a comparison of DT proportions across conditions was performed separately for the heads, bodies, and feet. The relationships between DT proportions to each IA and efficiency scores were also analysed to assess whether a focus on particular IAs was related to performance.

### Heads (head and neck)

**Fig 6:**
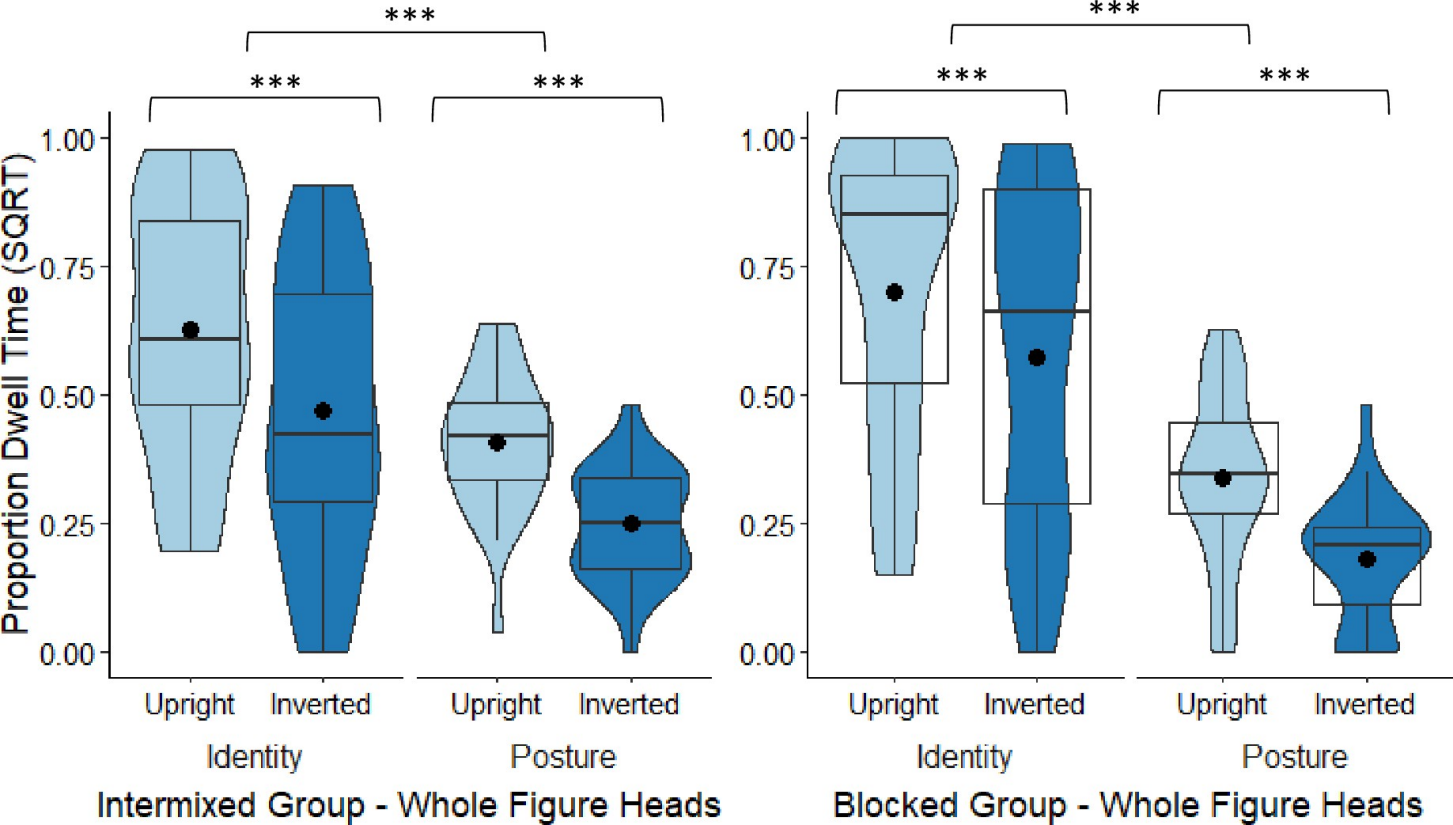
Intermixed and blocked groups’ box and violin plots of dwell time (DT) proportions (square-root transformed) to the heads in the whole figure upright and whole figure inverted conditions in the identity and posture tasks; dots denote means; *** = *p* < .001.

Only the whole figure conditions were included in this analysis. As the data were positively skewed, they were square-root transformed. This improved the distribution. A 2 × 2 × 2 mixed model ANOVA compared the square-root transformed DT proportions to the heads across the two groups (body types intermixed, body types blocked), between the two tasks (identity, posture), and between the two orientations (upright, inverted). The main effect of group was non-significant, but there was a main effect of task (Table 2). Original means are presented here for ease of interpretation, but the square root transformed data is presented in Fig 6. DT proportions to the heads were larger in the identity (*M* = 0.43, *SD* = 0.32) than in the posture task (*M* = 0.11, *SD* = 0.08). The main effect of orientation was also significant with greater looking at the heads of the whole figures in the upright (*M* = 0.34, *SD* = 0.22) compared to the inverted images (*M* = 0.21, *SD* = 0.18). The interaction between group and task was significant. To examine this, proportional DTs were averaged over the two orientations and the difference between the tasks was compared in each group. In the intermixed group, there was significantly greater looking at the heads in the identity (*M* = 0.37, *SD* = 0.27) than in the posture task (*M* = 0.13, *SD* = 0.07), *t*(27) = 5.48, *p* < .001, corrected *p* (α × 4) < .001, *d* = 1.04. This was also the case in blocked group (identity task: *M* = 0.50, *SD* = 0.34; posture task: *M* = 0.09, *SD* = 0.07), *t*(27) = 8.17, *p* < .001, corrected *p* (α × 4) < .001, *d* = 1.33. The interaction is likely explained by the larger effect size and mean difference between tasks in the blocked (*M* = 0.40, *SD* = 0.32) than in the intermixed group (*M* = 0.24, *SD* = 0.24), suggesting that the greater focus on heads in the identity task was stronger in the blocked group. The remaining interactions were non-significant (see Table 2 and Fig 6).

**Table 2:** Head IAs dwell time proportion comparisons.

Effect	
Group	*F*(1,53) = 0.04, *p* = .850, η_*p*_^2^ = .00
Task	***F*(1,53) = 96.48, *p* < .001, η**_***p***_^**2**^ **= .65**
Orientation	***F*(1,53) = 90.64, *p* < .001, η**_***p***_^**2**^ **= .63**
Group × task	***F*(1,53) = 6.64, *p* = .013, η**_***p***_^**2**^ **= .11**
Group × orientation	*F*(1,53) = 0.20, *p* = .653, η_*p*_^2^ = .00
Task × orientation	*F*(1,53) = 0.32, *p* = .574, η_*p*_^2^ = .01
Group × task × orientation	*F*(1,53) = 0.36, *p* = .549, η_*p*_^2^ = .01

The extra analyses with participant gender (see Supplementary Materials), revealed that the main effect of participant gender was non-significant, but there were significant group × gender, task × gender, and group × task × gender interactions (see Table B1 in S1 File). Follow-up simple effects analyses compared efficiency scores between female and male participants in the identity and posture tasks in the intermixed and blocked groups. In the intermixed group, the male participants looked at the heads in the identity task (with female images) more than the female participants (see Tables B2 and B3 in S1 File).

### Heads DT proportions summary

There was greater looking at heads in the identity than in the posture task and at the heads of the upright compared to the inverted whole figures. The greater focus on the heads in the identity than the posture task was stronger in the blocked than in the intermixed group, suggesting that when only seeing whole figures in a block, participants focus on the heads more than when seeing a mixture of headless and whole figures in a block. When it came to participant gender, male participants focussed more on the female heads in the identity task than female participants when the whole and headless figures were intermixed.

### Bodies (top of torso to ankles)

The data were significantly negatively skewed, so a reversed log transformation was performed. This did little to improve the distribution, so instead, any outliers (*z*-scores above 2 or below -2) were replaced with the means in each condition (intermixed identity task: 2 in HLU condition, 1 in HLI condition; intermixed posture task: 1 in WFU condition, 2 in WFI condition, 3 in HLU condition, 3 in HLI condition,; blocked identity task: 1 in HLU condition, 3 in HLI condition; blocked posture task: 2 in WFU condition, 2 in WFI condition, 3 in HLI condition, 2 in HLU condition). A 2 × 2 × 2 × 2 mixed model ANOVA comparing DT proportions to the bodies of the figures across the two groups (body types intermixed, body types blocked), between the two tasks (identity, posture), the two body types (whole figure, headless), and the two orientations (upright, inverted) revealed that the main effect of group was non-significant (see Table 3). However, there was a main effect of task. The DT proportions to bodies were significantly greater in the posture task (*M* = 0.87, *SD* = 0.09) than in the identity task (*M* = 0.77, *SD* = 0.17). The main effect of body type was also significant. DT proportions to the bodies were significantly larger in the headless (*M* = 0.95, *SD* = 0.05) than in the whole figure conditions (*M* = 0.69, *SD* = 0.21). The main effect of orientation was also significant with greater DT proportions to the inverted bodies (*M* = 0.84, *SD* = 0.13) than the upright bodies (*M* = 0.80, *SD* = 0.14).

**Table 3:** Body IAs dwell time proportion comparisons.

Effect	
Group	*F*(1,53) = 0.20, *p* = .664, η_*p*_^2^ = .00
Task	***F*(1,53) = 8.16, *p* = .006, η**_***p***_^**2**^ **= .13**
Body type	***F*(1,53) = 129.52, *p* < .001, η**_***p***_^**2**^ **= .71**
Orientation	***F*(1,53) = 22.07, *p* = .001, η**_***p***_^**2**^ **= .29**
Group × task	***F*(1,53) = 8.16, *p* = .006, η**_***p***_^**2**^ **= .13**
Group × body type	*F*(1,53) = 0.51, *p* = .478, η_*p*_^2^ = .01
Group × orientation	*F*(1,53) = 1.67, *p* = .202, η_*p*_^2^ = .03
Task × body type	***F*(1,53) = 78.19, *p* < .001, η**_***p***_^**2**^ **= .60**
Task × orientation	***F*(1,53) = 5.27, *p* = .026, η**_***p***_^**2**^ **= .09**
Body type × orientation	***F*(1,53) = 62.55, *p* < .001, η**_***p***_^**2**^ **= .54**
Group × task × body type	***F*(1,53) = 5.31, *p* = .025, η**_***p***_^**2**^ **= .09**
Group × task × orientation	*F*(1,53) = 0.22, *p* = .640, η_*p*_^2^ = .00
Group × body type × orientation	*F*(1,53) = 0.29, *p* = .865, η_*p*_^2^ = .00
Task × body type × orientation	*F*(1,53) = 2.04 *p* = .159, η_*p*_^2^ = .04
Group × task × body type × orientation	*F*(1,53) = 0.10, *p* = .756, η_*p*_^2^ = .00

The interactions between group and task, between task and body type, and between group, task, and body type were significant. To explore these interactions further, the DT proportions were averaged over orientations for each body type. As there was a main effect of body type (see above), the difference scores between DT proportions to the bodies of the whole figure and headless bodies were compared between each task type in each group. In the intermixed group, the difference in DT proportions between the headless and whole figure bodies was significantly larger in the identity task (*M* difference = 0.37, *SD* = 0.27) than in the posture task (*M* difference = 0.11, *SD* = 0.07), *t*(27) = 5.58, *p* < .001, corrected (α × 6) < .001, *d* = 1.06. That is, the greater looking at bodies of the headless figures over whole figures was stronger in the identity task than in the posture task. This was also the case in the blocked group: the greater looking at the bodies of the headless over whole figures was also significantly larger in the identity task (*M* difference = 0.49, *SD* = 0.33) than in the posture task (*M* difference = 0.05, *SD* = 0.08), *t*(27) = 6.82, *p* < .001, corrected *p* (α × 6) < .001, *d* = -1.31. The significant group by task by body type interaction is probably reflected by the larger effect size and difference score in the blocked than in the intermixed group. Therefore, when headless bodies appeared separately, participants had a particular focus on the bodies of the headless images in the identity task more so than in the posture task.

**Fig 7:**
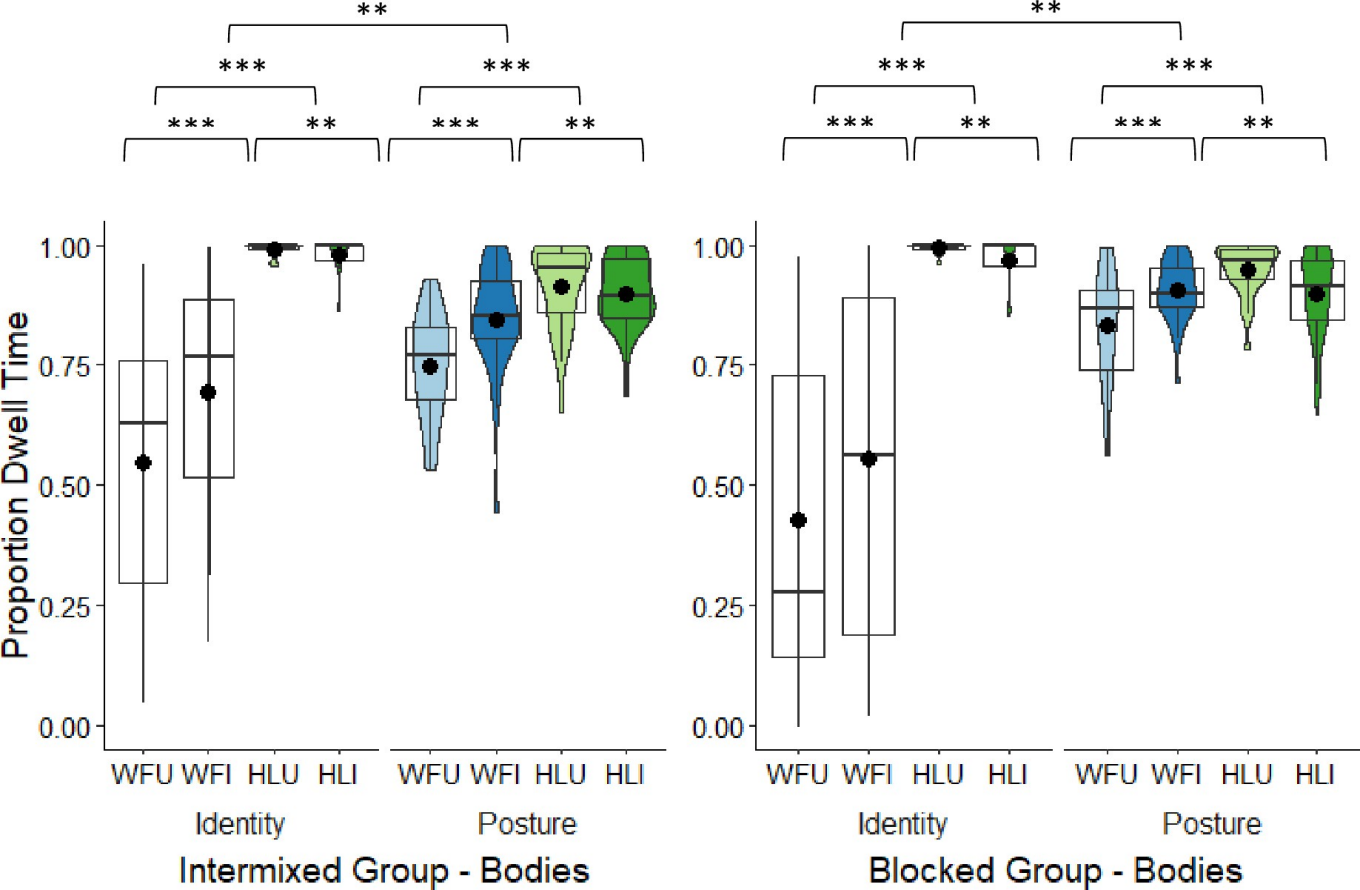
Intermixed and blocked groups’ box and violin plots of dwell time (DT) proportions (outliers removed) to the bodies in the whole figure upright (WFU), whole figure inverted (WFI), headless upright (HLU), and the headless inverted (HLI) conditions in the identity and posture tasks; dots denote means; *** = *p* < .001; ** = *p* < .01.

The interactions between task and orientation, and between body type and orientation were also significant. To explore these interactions further, DT proportions between upright and inverted figures were compared for each task and body type. In the identity task, the DT proportions to the bodies of the inverted whole figures (*M* = 0.62, *SD* = 0.30) were significantly larger than to the bodies of the upright whole figures (*M* = 0.49, *SD* = 0.33), *t*(55) = 5.53, *p* < .001, corrected (α × 6) < .001, *d* = 0.74; whereas for headless figures the DT proportions to the bodies of the upright headless figures (*M* = 0.99, *SD* = 0.01) were significantly larger than to the inverted headless figures (*M* = 0.97, *SD* = 0.04), *t*(55) = -3.66, *p* = .001, corrected (α × 6) = .006, *d* = -0.48. The posture task had the same pattern with significantly greater DT proportions to the bodies of the inverted whole figures (*M* = 0.87, *SD* = 0.10) than to the upright whole figures (*M* = 0.79, *SD* = 0.12), *t*(54) = 5.87, *p* < .001, corrected (α × 6) < .001, *d* = 0.79; and significantly greater DT proportions to the bodies of the upright headless figures (*M* = 0.93, *SD* = 0.08) than to the inverted headless figures (*M* = 0.90, *SD* = 0.08), *t*(55) = 2.89, *p* = .006, corrected (α × 6) = .036, *d* = 0.39. Therefore, in both tasks, for the whole figures, there was greater looking at bodies in the inverted than upright images, but the opposite was found with headless bodies with greater looking at the bodies in the upright compared to inverted images. The remaining interactions were non-significant (see Table 3 and Fig 7).

The extra analyses with participant gender (see Supplementary Materials) revealed no significant main effect of gender, but there were significant interactions between group × gender, task × gender, body type × gender, group × task × gender, group × body type × gender, task × body type × gender, and group × task × body type × gender (see Table C1 in S1 File). Comparisons were performed between female and males’ body dwell time proportions for each body type in each task separately for the two groups. This revealed that for the intermixed group, compared to male participants, female participants had larger dwell time proportions to the bodies of the whole figures in the identity task. There were no significant gender differences in the blocked study (see Tables C2 to C5 in S1 File).

### Body DT proportions summary

There was overall greater looking at the bodies in the posture than in the identity task. There was also greater looking at the bodies of headless figures than of the whole figures and this difference was larger in the identity task, presumably due to a greater focus on heads no longer possible with headless figures. With orientation, there was an interesting interaction in both the identity and posture tasks: for the whole figures, there was a greater focus on bodies in the inverted than the upright whole figures, but for the headless figures, there was greater focus on bodies in the upright than inverted figures. This is likely due to a greater focus on heads than bodies in the whole figures particularly when upright, and a greater focus on bodies when the figures are headless. When it came to participant gender, female participants in the intermixed group looked more than male participants to the bodies of the whole figures in the identity task, which involved female images.

### Feet (from ankles and bottom of feet)

**Fig 8:**
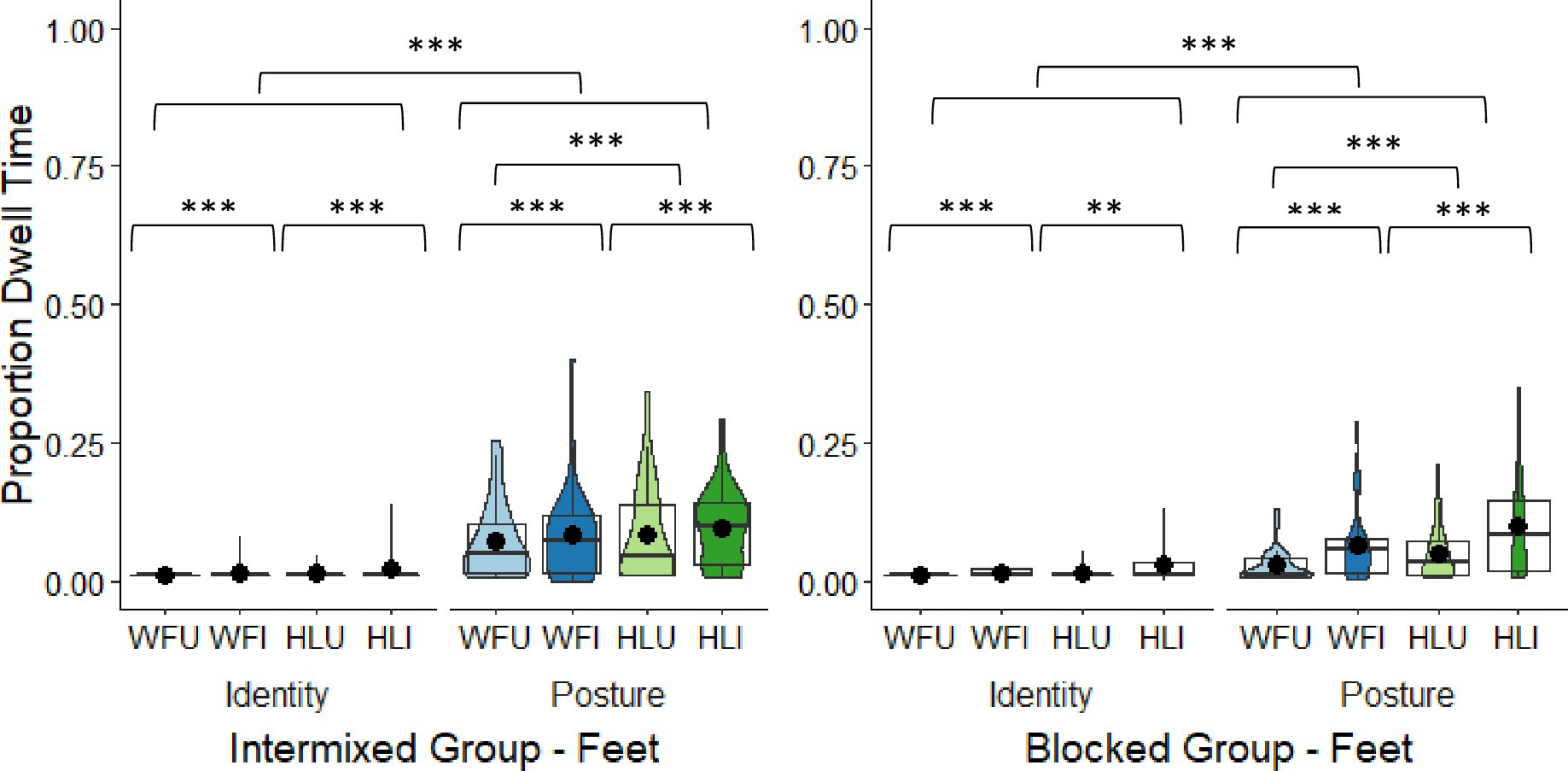
Intermixed and blocked groups’ box and violin plots of dwell time (DT) proportions to the feet in the whole figure upright (WFU), whole figure inverted (WFI), headless upright (HLU), and the headless inverted (HLI) conditions in the identity and posture tasks; dots denote means; *** = *p* < .001; ** = *p* < .01.

The data were significantly positively skewed. A log transformation did little to improve the distribution, so outliers (*z*-scores above 2 or below -2) were replaced with the means in each condition which improved the distribution (intermixed identity task: 4 in WFU condition, 3 in WFI condition, 1 in HLU condition, 2 in HLI condition; intermixed posture task: 2 in WFU condition, 1 in WFI condition, 3 in HLI condition, 3 in HLU condition; blocked identity task: 4 in WFU condition, 3 in WFI condition, 1 in HLU condition, 2 in HLI condition; blocked posture task: 2 in WFU condition, 2 in WFI condition, 3 in HLI condition, 2 in HLU condition). A 2 × 2 × 2 × 2 mixed model ANOVA comparing DT proportions to feet across the two groups (body types intermixed, body types blocked), between the two tasks (identity, posture), the two body types (whole figure, headless), and the two orientations (upright, inverted) revealed that the main effect of group was non-significant (see Table 4 and Fig 8). The main effect of task was significant as DT proportions overall greater to the feet in the posture (*M* = 0.07, *SD* = 0.07) than in the identity task (*M* = 0.02, *SD* = 0.01). This might be due to greater variability in the feet positions in the posture than in the identity task. The main effect of body type was also significant. DT proportions were overall greater to the feet in the headless (*M* = 0.05, *SD* = 0.05) than in the whole figure conditions (*M* = 0.04, *SD* = 0.04). The main effect of orientation was also significant. DT proportions were overall greater to the feet in the inverted (*M* = 0.05, *SD* = 0.05) than in the upright conditions (*M* = 0.04, *SD* = 0.04). The task by orientation interaction was significant. To explore this, DT proportions to feet were averaged over body types and the DT proportions were compared between orientations in each task using Bonferroni post-hoc comparisons. In the identity task, there was greater looking at the feet in the inverted (*M* = 0.02, *SD* = 0.02) compared to the upright condition (*M* = 0.01, *SD* = 0.01), *t*(55) = 2.63, *p* = .011, corrected (α × 2) = .022, *d* = 0.35. In the posture task, there was also greater looking at the feet in the inverted (*M* = 0.07, *SD* = 0.07) compared to the upright condition (*M* = 0.06, *SD* = 0.06), *t*(55) = 2.84, *p* = .006, corrected (α × 2) = .012, *d* = 0.38. The remaining interactions were all non-significant (see Table 4).

**Table 4:** Feet IAs dwell time proportion comparisons.

Effect	
Group	*F*(1,53) = 2.13, *p* = .150, η_*p*_^2^ = .04
Task	***F*(1,53) = 65.94, *p* < .001, η**_***p***_^**2**^ **= .55**
Body type	***F*(1,53) = 13.55, *p <* .001, η**_***p***_^**2**^ **= .20**
Orientation	***F*(1,53) = 12.73, *p* < .001, η**_***p***_^**2**^ **= .19**
Group × task	*F*(1,53) = 2.97, *p* = .091, η_*p*_^2^ = .05
Group × body type	*F*(1,53) = 1.59, *p* = .213, η_*p*_^2^ = .03
Group × orientation	*F*(1,53) = 2.79, *p* = .101, η_*p*_^2^ = .05
Task × body type	*F*(1,53) = 2.65, *p* = .110, η_*p*_^2^ = .05
Task × orientation	***F*(1,53) = 4.26, *p* = .044, η**_***p***_^**2**^ **= .07**
Body type × orientation	*F*(1,53) = 1.00, *p* = .323, η_*p*_^2^ = .02
Group × task × body type	*F*(1,53) = 0.41, *p* = .525, η_*p*_^2^ = .01
Group × task × orientation	*F*(1,53) = 2.05, *p* = .158, η_*p*_^2^ = .04
Group × body type × orientation	*F*(1,53) = 0.72, *p* = .400, η_*p*_^2^ = .01
Task × body type × orientation	*F*(1,53) = 0.00, *p* = .962, η_*p*_^2^ = .01
Group × task × body type × orientation	*F*(1,53) = 0.00, *p* = .973, η_*p*_^2^ = .00

The analyses involving participant gender revealed a significant main effect of gender with female participants looking at the feet more than the male participants. There was also a significant task × gender interaction (see Table D1 in S1 File). Female and males’ dwell time proportions to feet were compared in each task. Compared to male participants, female participants had larger dwell time proportions to the feet in the posture task. There was no significant gender difference in the identity task (see Tables D2 and D3 in S1 File).

### Feet DT proportions summary

There was greater looking at the feet in the posture than in the identity task possibly due to greater variability in feet positions in the posture task. There was also greater looking at feet in the headless than in the whole figures. As expected, there was also overall greater looking at the feet in the inverted than the upright images and this was the case in both the identity and posture tasks. In the absence of heads (headless figures) or when feet were in the upper regions (inverted images), there was more attention directed towards the feet. Considering participant gender, female participants looked longer at feet than male participants in the posture task, which involved male feet.

### Relationship between DT proportions and performance

To see whether the DT proportions to the heads, bodies, or feet were associated with performance, Spearman’s ρ correlations were performed between the DT proportions to each IA and efficiency scores (see Table 5). As the main effect of group was non-significant in the DT proportion analyses above, we combined the two groups, but performed separate analyses for each condition and for the two tasks. For the whole figures, Bonferroni corrections were applied based on the presence of three IAs: heads, bodies, feet (α × 3). For the headless images, Bonferroni corrections were based on the presence of two IAs: bodies and feet (α × 2).

**Table 5:** Spearman’s ρ correlations between efficiency scores and proportional dwell time to heads, bodies, and feet.

Identity Task	Dwell Time (DT) Proportion			Posture Task	Dwell Time (DT) Proportion		
	Heads	Bodies	Feet		Heads	Bodies	Feet
Whole Figure Upright				Whole Figure Upright			
Spearman’s ρ	.083	-.079	-.050	Spearman’s ρ	**.488**	**-.544**	**.343**
(*p*-value)	(.543)	(.561)	(.717)	(*p*-value)	**(< .001)**	**(< .001)**	**(.010)**
corrected p*	(.999)	(.999)	(.999)	corrected p*	**(< .001)**	**(< .001)**	**(.030)**
Whole Figure Inverted				Whole Figure Inverted			
Spearman’s ρ	.191	-.181	-.087	Spearman’s ρ	**.501**	**-.516**	**.440**
(*p*-value)	(.160)	(.182)	(.524)	(*p*-value)	**(< .001)**	**(< .001)**	**(< .001)**
corrected p*	(.480)	(.546)	(.999)	corrected p*	**(< .001)**	**(< .001)**	**(< .001)**
Headless Upright				Headless Upright			
Spearman’s ρ	—	-.074	.000	Spearman’s ρ	—	-.232	.221
(*p*-value)		(.586)	(.998)	(*p*-value)		(.086)	(.102)
corrected p*		(.999)	(.999)	corrected p*		(.172)	(.204)
Headless Inverted				Headless Inverted			
Spearman’s ρ	—	-.135	-.048	Spearman’s ρ	—	**-.341**	**.344**
(*p*-value)		(.319)	(.725)	(*p*-value)		**(.010)**	**(.010)**
corrected p*		(.638)	(.999)	corrected p*		**(.020)**	**(.030)**

*Bonferroni corrections (α × 3 for each whole figure conditions and α × 2 for each headless conditions)

### Whole figure upright images

In the identity task, there were no significant relationships between efficiency scores and DT proportions to any of the IAs. However, in the posture task, there was a significant positive relationship between efficiency scores and DT proportions to the heads and feet, and a negative relationship between efficiency scores and DT proportion to the bodies (see Table 5). Therefore, greater looking at the heads and feet of the upright whole figures was associated with poorer efficiency, but greater looking at the bodies was associated with better efficiency in the posture condition.

### Whole figure inverted images

In the identity task, relationships between efficiency scores and DT proportions to the IAs were again non-significant. However, in the posture task efficiency scores were positively related to DT proportions to the heads and feet and negatively related to DT proportion to the bodies. Therefore, during posture discrimination, greater looking at the heads and feet of the inverted whole figures was associated with poorer efficiency scores, but greater looking at the bodies was associated with better efficiency, giving the same pattern as for upright whole figures.

### Headless upright images

In both the identity and posture task, there were no significant relationships between DT proportions to any of the IAs and efficiency scores (following Bonferroni corrections).

### Headless inverted images

In the identity task, there were no significant relationships between DT proportions and efficiency scores. In the posture task, there was a significant positive relationship between DT proportions to the feet and efficiency scores, and a negative relationship between DT proportions to the bodies and efficiency scores.

### Summary of relationship between performance and DT proportions

In the identity task, there were no significant relationships between DT proportions and performance. In the posture task, the more participants looked at heads and feet the poorer the efficiency scores, but the more they looked at the bodies the better the efficiency scores. This was the case for all conditions aside from the upright headless condition. This suggests that in posture discrimination a focus on bodies is associated with better performance, but this is not the case in identity discrimination.

## General discussion

The aim of the current study was to investigate potential reasons for inconsistent headless BIEs in the literature. A key question was whether the faces or implied faces of the heads of whole figures contributes to configural processing in headless bodies due to repetition priming [10, 12]. In previous studies, when participants saw only headless figures, headless BIEs were not found [1, 12], but in other studies, headless BIEs were found [13, 14]. Similarly, when participants saw both whole and headless figures in the same blocks, headless BIEs were also inconsistent across studies [2, 10, 16]. Another question is whether the inconsistent headless BIEs are due to the task involving identity or posture discrimination [1, 2, 13, 16]. Here, we compared whole and headless BIEs within groups, but compared the possible effects of priming by comparing a group in which the whole and headless figures were intermixed to a group where they were blocked with headless figures presented first to prevent priming. In both groups, BIEs were found for whole and headless figures and for both tasks. Participants were more efficient in discriminating pairs of upright bodies than pairs of inverted bodies and this was regardless of whether the bodies had heads or the task involved discriminating identities or postures. Importantly, BIEs were also found regardless of whether participants saw headless figures in the same blocks or separately to whole figures. Headless BIEs have been found in posture tasks before [10, 13, 14], but Robbins and Coltheart [2] is the only other known study to have found a headless BIE in a body identity discrimination task.

It was also expected, like previous findings, that the headless BIEs would be weaker in magnitude than whole figure BIEs [2, 13]. In the blocked group, where whole and headless figures appeared separately, the overall magnitude of the BIE was larger for the whole than for the headless figures. This was not the case when the presentation of whole and headless bodies was intermixed, which suggests that when whole and headless figures appeared in the same blocks, the heads of the whole figures possibly enhanced the headless BIEs. Further analyses revealed that the difference in BIE magnitude between whole and headless figures was only in the identity, but not the posture task in both groups. This is likely due to the presence of faces in the identity task contributing to participants’ ability to discriminate upright whole figures. Overall the findings suggests that heads play an influential role in BIEs, but as BIEs were found with whole and headless figures in both groups and in identity and posture discrimination, heads are not essential.

The eye tracking data revealed that proportional dwell times (DTs) to the heads were greater for the upright compared to the inverted figures in both tasks,. Therefore there was a stronger focus on heads in their typical orientation. There was also a greater focus on heads in the identity than the posture task and this was stronger in the blocked group where whole figures appeared separately to the headless images. Thus, when whole figures appear alone, the focus is centred on heads. There was also a greater focus on bodies in the headless compared to the whole figures and this difference was stronger in the identity than in the posture task. The greater focus on bodies in the headless identity condition was also stronger in the blocked group where participants saw headless images on their own. Presenting whole and headless bodies separately seems to lead to a stronger focus on heads in the whole figures and bodies in the headless conditions. There was also an overall greater focus on bodies and feet in the posture than in the identity task. Performance was better in the identity than the posture task, but despite this and the differences in proportional DTs to heads and bodies between the identity and posture tasks, the magnitude of the BIEs between the tasks did not differ significantly for the whole and headless figures. Therefore, when whole and headless figures appeared separately, participants likely focussed on the features they thought were most informative, but this did not lead to a difference in BIE magnitude across the posture and identity tasks.

For both tasks, there was also an interesting interaction between body type and orientation: for the whole figures there was greater looking at the bodies of the inverted images than the bodies of the upright images, but for the headless figures there was greater looking at bodies of the upright images than the bodies of the inverted images. Therefore, when heads are missing, participants focus on the bodies particularly when they are upright, but when heads are available, they focus more on bodies when they are inverted. This is presumably due to greater looking at the heads of upright whole figures. There was also a greater focus on feet in the inverted over the upright figures, and a greater focus on feet in the headless than in the whole figures. As in Arizpe et al.’s study [13], there is an upper bias in posture discrimination, and a focus on lower parts of the inverted figures. In sum, these findings suggest that participants focus on heads when they are available and upright, but they focus on bodies and feet when the figures are headless or inverted.

A greater focus on bodies does not necessarily correspond with better body discrimination. In previous studies of posture discrimination, participants focussed more on the upper regions of the upright figures (heads and upper torso) and lower regions of inverted figures, but there was better performance with upright figures [10, 13]. Arizpe et al. [13] found better performance when participants were instructed to focus on the upper regions compared to when instructed to focus on lower regions, but this was not the case with headless bodies. Arizpe et al. [13] argued that BIEs are likely based on orientation-specific configural processing of bodies and less on low-level part-based processing; otherwise there would be better performance when participants focussed on limb positions. Arizpe et al. [13] further argued that due to a lack of a gaze location effect with the headless stimuli, orientation-specific configural processing driving a headless BIE might differ slightly to the processing of whole figures. The greater focus on the bodies of upright headless images (compared to upright whole figure bodies) found here and previously [10], might involve a combination of configural and part-based processing in the headless BIE. Axelsson et al. [10] found an association between greater looking at inverted bodies and better performance suggesting that with inverted bodies, discrimination was likely aided by low-level body part processing. Therefore, where participants look and its association with performance varies.

Based on the above findings, it was predicted here that a focus on heads would be associated with better performance with upright whole figures, and a greater focus on bodies would be associated with better performance with inverted images. In the identity task, the correlations between efficiency scores and DT proportions to the heads, bodies, and feet were all non-significant. In the posture task, in all conditions aside from the upright headless condition (following Bonferroni corrections), greater proportional looking to the bodies and less to the heads and feet was associated with better performance. The differing findings across the two tasks could be due to greater variability in limb positions in the posture task than the identity task, or smaller range of scores in the identity task than the posture task (see Figs 4 and 5). In the identity task, despite the non-significant correlations, performance was better in the identity than in the posture task, yet there was a greater focus on heads and less on bodies in the identity task. Therefore, as with previous studies [e.g., 10, 13], a focus on bodies does not always reflect better performance. Unlike here, Arizpe et al. [13] found that a focus on heads was associated with better performance in posture discrimination. This might be due to the heads of the pairs here being in identical positions whereas in Arizpe et al. the head positions varied. In sum, the findings here suggest that where participants look and associations with performance varies by task type.

### Do the differences in study characteristics explain the inconsistent headless BIEs?

Previous studies differed in several ways, and we compared the task type (posture versus identity discrimination), and the study design (whole and headless figures presented together or separately). When considering task type, despite better overall performance in the identity compared to the posture task, BIEs were found in both and the magnitude of the BIE did not differ across tasks in both whole and headless body discrimination. Therefore, task type is an unlikely explanation for the inconsistent headless BIEs in the literature.

The key experimental manipulation here was study design, and whether participants saw whole and headless stimuli together or separately as this is what differed in previous studies. Here, headless BIEs were found whether headless figures were presented with or without whole figures. However, when whole and headless figures appeared separately (blocked), the BIE magnitude was overall larger for the whole compared to the headless figures, but not when they appeared in the same blocks (intermixed). It suggests that seeing the heads of the whole figures in the same trial block might lead to slightly stronger headless BIEs. Later planned contrasts revealed that the difference in BIE magnitude between the whole and headless figures was only in the identity, but not the posture task. This is likely due to the differing heads in the pairs of whole figures in the identity task.

### Limitations

The main aim was to investigate headless BIEs, but a limitation was the inclusion of faces in the whole figures. This led to a difference between the identity and posture task. The individuals in the pairs in the posture task were identical aside from the limb positions. The individuals in the pairs in the identity task were different, and participants likely used face and body information to discriminate. There was a greater focus on heads in the identity over the posture task and better performance. Therefore, it is unclear whether participants are better at identity over posture discrimination and future studies should ideally erase facial information. However, the key focus was on the headless images and whether the study design affects headless BIEs. The task by body type interaction was non-significant suggesting that participants did not perform better overall at discriminating the whole and headless bodies in either task. The relationship between looking at heads and efficiency was also non-significant in the identity task. Therefore, it is unclear that faces in the identity task led to better performance, but faces should be removed in future studies.

Another possible limitation is that the head positions of the whole figure pairs were identical in the posture task. The aim was to see whether head and facial information of the whole figures rather than their positions would influence headless BIEs. However, as stronger BIEs have been found with pairs of whole figures with varied than with fixed head positions previously [1], this might explain the lack of difference in BIE magnitude between the whole and headless figures in the posture task. Yet, Axelsson et al. [10] used the same stimuli with identical head positions, and the effect sizes for the whole figures were larger than they were for the headless figures. Nonetheless, it would be useful to compare varied and fixed head positions between whole and headless conditions to determine the role of heads separately to faces.

Another limitation is that the stimuli in the identity task were female and the stimuli in the posture task were male. This is because the stimuli were created separately for separate studies [2, 10] and we wanted to be able to compare the results. One issue is whether there are differences in body-related cues provided by each gender. Are there features of the genders that more greatly distinguish individuals within pairs? We attempted to match overall body shape within the pairs, but future studies could test whether gender of the stimuli contributes to differences body discrimination. Another question is whether gender of the participants affected performance and looking times. There is some evidence of better face recognition in females, but no differences in configural processing as measured by inversion effects [e.g., 22]. Extra analyses were performed here, but these results should be interpreted with caution due to the small number of male participants and unequal numbers of male and female participants. Sexuality data was also not collected. For the efficiency scores, male participants in the blocked group were more efficient than female participants in the posture task (see Tables A1 and A3 in S1 File). The posture task involved male bodies, so perhaps seeing bodies of the same gender aided in their performance when the whole and headless bodies appeared in separate blocks. However, participant gender did not interact with orientation or body type the key variables of interest here.

For the head dwell time proportions, there was a significant group by task by gender interaction (see Table B5 in S1 File). This was due to the male participants looking at the heads in the identity task more than the female participants (see Tables B1 and B3 in S1 File). The identity task involved female participants, and the intermixed group saw a mixture of whole and headless figures. The male participants were possibly making greater use of the features from the heads when they were available.

For the body dwell time proportions, there were significant interactions involving the variables group, task, body type, and gender (see Table C1 in S1 File). This was due to females in the intermixed group looking longer than male participants to the bodies of the whole figures in the identity task (see Tables C2 to C5 in S1 File). The identity task contained female images. Perhaps females took more interest in bodies of the same gender.

For the feet dwell time proportions, female participants looked at the feet more than the male participants particularly in the posture task (see Tables D1 to D3 in S1 File). The posture task involved male images, but the posture task also involved more variable feet positions and female participants made up the majority of the sample. This result might also reflect the finding above that male participants were more focused on the male bodies in the posture task reducing the time spent looking at feet.

Previous studies have found an effect of gender of the stimuli with an absence of a BIE with sexualised female bodies, which they interpreted as showing that female bodies are less configurally processed or “objectified” [e.g., 22]. However, cautions have been raised such as the task being mirror reversal rather than visual processing of bodies [see 23, 24]. More recently, Cazzato et al. [25] found no effect of participant gender and found BIEs for both female and male stimuli, but with better processing of inverted male bodies. With the findings here, there are perhaps effects of embodiment or greater identification or perceptual sensitivity with participants focusing more on bodies of the same gender. Bidet-Ildei and Bouquet [26] found with dynamic displays of people running, that male participants were significantly slower in judging the running direction of a centrally-located target runner, when it was flanked by male stimuli running in the opposite compared to the same direction. Responses were faster if the male flankers were running the same direction as the central target. This resembles the findings here as male participants in the blocked group were more efficient than female in the posture task which involved discriminating male bodies. Pavlova et al. [27] found with point-light displays (PLDs—displays with no bodily features, instead showing only movement of lights on the major joints and head), earlier activation in female than male participants in parietal and temporal regions. Male participants had greater activations later in the frontal and occipital regions. Therefore, there are possibly gender differences in the earlier detection and later cognitive processing of bodies. Future tasks should involve male and female stimuli and have equal numbers of male and female participants.

Another issue to consider is that the stimuli here were static, and bodies are typically moving. Studies with PLDs have revealed that despite the speed of recognition of human motion with upright displays, inversion, even from a 90° rotation, dramatically disrupts body detection of walking figures [28, 29]. Piepers et al. [30] also found similar inversion effects for moving and static faces and bodies where the features are visible. The inversion effects were larger for human than for moving and static dogs. In identity discrimination, Simhi and Yovel [31] found that discrimination of headless bodies was better in dynamic than in static displays. They also found no difference in discrimination performance between headless and whole figures in dynamic displays, but participants were worse at discriminating headless bodies in static displays. Therefore, heads play an important role in body identity discrimination in static, but not dynamic displays. It is likely that processing of headless bodies benefits from both form and motion information [see also 32]. Interestingly, the extrastriate and fusiform body areas both respond to form and body motion [33, 34]. However, observers are still sensitive to differences in static displays of bodies. Robbins and Coltheart [35] found that heads aided in identity discrimination, but that the inclusion of movement did little to boost performance.

Despite better performance in the identity than the posture task here, it is likely that judgements of actions or intentions from postures would also be improved in dynamic contexts. Here, there was a greater focus on the feet in the posture than the identity task. PLD studies have revealed the importance of feet in dynamic stimuli, which is likely because the largest degree of movement involves the feet [36]. Inversion disrupts configural processing, but future studies could employ dynamic stimuli to investigate the role of key features such as heads and feet in body inversion effects in identity and posture tasks.

A final consideration is a perceptual difference between the whole and headless figures–there is a greater amount of light areas in the headless images. Both the upright and inverted conditions had the same manipulation, with the presence and absence of a head, and it is the size of inversion effects for whole figures and headless that was of interest. However, one way to deal with the size of the lighter areas in the images is to include a condition where the heads in the headless images are replaced by an average head-sized and head-coloured square. Robbins and Coltheart [35] covered up heads with hats instead of removing them, and they found that identity recognition was poorer when heads were covered rather than the bodies, further indicating the importance of heads in identity recognition. However, the inclusion of an object in the place of heads could also disrupt the visual processing of bodies in other ways. Another option is to include the brightness and image size variables as covariates in the analyses to determine if they interact with performance.

## Conclusion

If headless BIEs are only induced by priming after seeing the heads of whole figures, we should have found no BIEs in the blocked group. Instead BIEs were found in all conditions. As headless BIEs were found in identity and posture discrimination and when participants saw headless figures presented with and without whole figures, task type and repetition priming alone do not explain previous inconsistent BIEs. However, there were some differences between the intermixed and blocked study suggesting that repetition priming of heads might play a subtle role in headless BIEs. With sufficient sample sizes, headless BIEs are found [2, 10, 13–15], but the heads and implied faces of forward facing figures might play a role [10, 12]. A remaining question is why headless BIEs are sometimes absent or weaker. Regardless, multiple studies have found headless BIEs and they are likely based on configural processing [2, 10, 13, 14]. Axelsson et al. [10] argued that BIEs are likely explained by better discrimination performance with more typical figures, which in the case here refers to being, upright, whole, and forward-facing. Inconsistent headless BIEs might reflect processing changing as a result of viewing atypical stimuli when heads are absent. Faces might contribute to a BIE [12], but they are not essential. A shift from focussing on the heads of whole figures to focussing on the bodies of upright headless figures suggests that the configural processing of whole figure and headless bodies might differ slightly [see 13]. Headless BIEs are likely based on configural as well as featural processing.

## Supporting information

S1 File (DOCX)
