# Supplementary material for: Making heads or tails of body inversion effects: Do heads matter?
Source: PLoS One. 2022 Feb 17;17(2):e0263902. doi: 10.1371/journal.pone.0263902 (PMC8853516; doi:10.1371/journal.pone.0263902)
Supplement: S1 File — (DOCX) [file pone.0263902.s001.docx]

Supplementary Materials

[**Gender of Participant Analyses** 3](#_Toc87878161)

[**Efficiency Scores with Participant Gender** 3](#_Toc87878162)

[**Table A1** 3](#_Toc87878163)

[*Efficiency Scores: Fixed Effects Parameter Estimates for the Linear Mixed Effects Model Analysis Including Gender* 3](#_Toc87878164)

[**Table A2** 5](#_Toc87878165)

[*Follow-up Efficiency Score Comparison Between Participant Genders for Tasks in the Intermixed and Blocked Groups* 5](#_Toc87878166)

[**Table A3** 5](#_Toc87878167)

[*Efficiency Scores for Females and Males for the Tasks in the Intermixed and Blocked Groups* 5](#_Toc87878168)

[**Head Interest Area Analyses with Participant Gender** 6](#_Toc87878169)

[**Table B1** 6](#_Toc87878170)

[*Head DT Proportions (Square-root Transformed): Fixed Effects Parameter Estimates for the Linear Mixed Effects Model Analysis Including Participant Gender* 6](#_Toc87878171)

[**Table B2** 7](#_Toc87878172)

[*Follow-up Head Dwell Time Proportions (Square-root Transformed) Comparison Between Participant Genders for Tasks in the Intermixed and Blocked Groups* 7](#_Toc87878173)

[**Table B3** 7](#_Toc87878174)

[*Head Dwell Time Proportions (Square-root Transformed) for Females and Males for the Tasks in the Intermixed and Blocked Groups* 7](#_Toc87878175)

[**Body Interest Area Analyses with Participant Gender** 8](#_Toc87878176)

[**Table C1** 8](#_Toc87878177)

[*Body Dwell Time Proportions (Outliers Removed): Fixed Effects Parameter Estimates for the Linear Mixed Effects Model Analysis Including Participant Gender* 8](#_Toc87878178)

[**Table C2** 10](#_Toc87878179)

[*Follow-up Body Dwell Time Proportions Comparison Between Participant Genders for Body Types in Each Task in the Intermixed Group* 10](#_Toc87878180)

[**Table C3** 10](#_Toc87878181)

[*Body Dwell Time Proportions (Outliers Removed) for Females and Males for the Body Types in Each Task in the Intermixed Group* 10](#_Toc87878182)

[**Table C4** 11](#_Toc87878183)

[*Follow-up Body Dwell Time Proportions Comparison Between Participant Genders for Body Types in Each Task in the Blocked Group* 11](#_Toc87878184)

[**Table C5** 11](#_Toc87878185)

[*Body Dwell Time Proportions (Outliers Removed) for Females and Males for the Body Types in Each Task in the Blocked Group* 11](#_Toc87878186)

[**Feet Interest Area Analyses with Gender** 12](#_Toc87878187)

[**Table D1** 12](#_Toc87878188)

[*Feet Dwell Time Proportions (Outliers Removed): Fixed Effects Parameter Estimates for the Linear Mixed Effects Model Analysis Including Participant Gender* 12](#_Toc87878189)

[**Table D2** 14](#_Toc87878190)

[*Follow-up Feet Dwell Time Proportions Comparison Between Genders in Each Task* 14](#_Toc87878191)

[**Table D3** 14](#_Toc87878192)

[*Feet Dwell Time Proportions (Outliers Removed) for Females and Males in Each Task* 14](#_Toc87878193)

# **Gender of Participant Analyses**

Given that the identity task involved female images and the posture task involved male images, the analyses from the main manuscript were performed again with participant gender to determine if they responded differently to each other. These were analysed with linear mixed effects models in jamovi 2 software, using the GAMLj module 2.0.1 (2019), a module developed in R (2019) with the lme4 package (Bates et al., 2015).

# **Efficiency Scores with Participant Gender**

## **Table A1**

## *Efficiency Scores: Fixed Effects Parameter Estimates for the Linear Mixed Effects Model Analysis Including Gender*

|  | | | | | | | | 95% Confidence Interval | | | |  | | | | | |
| --- | --- | --- | --- | --- | --- | --- | --- | --- | --- | --- | --- | --- | --- | --- | --- | --- | --- |
| Names | | Effect | | Estimate | | *SE* | | Lower | | Upper | | *df* | | *t* | | *p* | |
| (Intercept) |  | (Intercept) |  | 1408.80 |  | 40.68 |  | 1329.06 |  | 1488.54 |  | 56 |  | 34.63 |  | < .001 |  |
| group |  | blocked - intermixed |  | -140.68 |  | 81.37 |  | -300.16 |  | 18.80 |  | 56 |  | -1.73 |  | .089 |  |
| **task** |  | **posture - identity** |  | **525.09** |  | **24.72** |  | **476.64** |  | **573.54** |  | **392** |  | **21.24** |  | **< .001** |  |
| **body type** |  | **whole figure - headless** |  | **-98.19** |  | **24.72** |  | **-146.64** |  | **-49.74** |  | **392** |  | **-3.97** |  | **< .001** |  |
| **orientation** |  | **upright - inverted** |  | **-231.65** |  | **24.72** |  | **-280.10** |  | **-183.20** |  | **392** |  | **-9.37** |  | **< .001** |  |
| gender |  | male - female |  | -3.85 |  | 81.37 |  | -163.33 |  | 155.63 |  | 56 |  | -0.05 |  | .962 |  |
| **group ✻ task** |  | **blocked - intermixed ✻ posture - identity** |  | **-202.92** |  | **49.44** |  | **-299.82** |  | **-106.02** |  | **392** |  | **-4.10** |  | **< .001** |  |
| **group ✻ body type** |  | **blocked - intermixed ✻ whole figure - headless** |  | **-202.51** |  | **49.44** |  | **-299.41** |  | **-105.61** |  | **392** |  | **-4.10** |  | **< .001** |  |
| task ✻ body type |  | posture - identity ✻ whole figure - headless |  | -67.04 |  | 49.44 |  | -163.94 |  | 29.86 |  | 392 |  | -1.36 |  | .176 |  |
| group ✻ orientation |  | blocked - intermixed ✻ upright - inverted |  | 7.34 |  | 49.44 |  | -89.56 |  | 104.24 |  | 392 |  | 0.15 |  | .882 |  |
| task ✻ orientation |  | posture - identity ✻ upright - inverted |  | 7.12 |  | 49.44 |  | -89.78 |  | 104.02 |  | 392 |  | 0.14 |  | .886 |  |
| body type ✻ orientation |  | whole figure - headless ✻ upright - inverted |  | -96.53 |  | 49.44 |  | -193.43 |  | 0.37 |  | 392 |  | -1.95 |  | .052 |  |
| **group ✻ gender** |  | **blocked - intermixed ✻ male - female** |  | **-332.53** |  | **162.74** |  | **-651.49** |  | **-13.57** |  | **56** |  | **-2.04** |  | **.046** |  |
| task ✻ gender |  | posture - identity ✻ male - female |  | -88.18 |  | 49.44 |  | -185.08 |  | 8.72 |  | 392 |  | -1.78 |  | .075 |  |
| body type ✻ gender |  | whole figure - headless ✻ male - female |  | 23.21 |  | 49.44 |  | -73.69 |  | 120.11 |  | 392 |  | 0.47 |  | .639 |  |
| orientation ✻ gender |  | upright - inverted ✻ male - female |  | 60.05 |  | 49.44 |  | -36.85 |  | 156.95 |  | 392 |  | 1.21 |  | .225 |  |
| group ✻ task ✻ body type |  | blocked - intermixed ✻ posture - identity ✻ whole figure - headless |  | -143.42 |  | 98.88 |  | -337.22 |  | 50.38 |  | 392 |  | -1.45 |  | .148 |  |
| group ✻ task ✻ orientation |  | blocked - intermixed ✻ posture - identity ✻ upright - inverted |  | -34.26 |  | 98.88 |  | -228.06 |  | 159.54 |  | 392 |  | -0.35 |  | .729 |  |
| group ✻ body type ✻ orientation |  | blocked - intermixed ✻ whole figure - headless ✻ upright - inverted |  | -1.03 |  | 98.88 |  | -194.82 |  | 192.77 |  | 392 |  | -0.01 |  | .992 |  |
| task ✻ body type ✻ orientation |  | posture - identity ✻ whole figure - headless ✻ upright - inverted |  | 115.75 |  | 98.88 |  | -78.05 |  | 309.55 |  | 392 |  | 1.17 |  | .242 |  |
| **group ✻ task ✻ gender** |  | **blocked - intermixed ✻ posture - identity ✻ male - female** |  | **-314.22** |  | **98.88** |  | **-508.02** |  | **-120.42** |  | **392** |  | **-3.18** |  | **.002** |  |
| group ✻ body type ✻ gender |  | blocked - intermixed ✻ whole figure - headless ✻ male - female |  | 7.39 |  | 98.88 |  | -186.41 |  | 201.19 |  | 392 |  | 0.07 |  | .940 |  |
| task ✻ body type ✻ gender |  | posture - identity ✻ whole figure - headless ✻ male - female |  | 42.65 |  | 98.88 |  | -151.15 |  | 236.45 |  | 392 |  | 0.43 |  | .666 |  |
| group ✻ orientation ✻ gender |  | blocked - intermixed ✻ upright - inverted ✻ male - female |  | 112.60 |  | 98.88 |  | -81.20 |  | 306.40 |  | 392 |  | 1.14 |  | .255 |  |
| task ✻ orientation ✻ gender |  | posture - identity ✻ upright - inverted ✻ male - female |  | 112.72 |  | 98.88 |  | -81.08 |  | 306.51 |  | 392 |  | 1.14 |  | .255 |  |
| body type ✻ orientation ✻ gender |  | whole figure - headless ✻ upright - inverted ✻ male - female |  | -91.49 |  | 98.88 |  | -285.29 |  | 102.31 |  | 392 |  | -0.93 |  | .355 |  |
| group ✻ task ✻ body type ✻ orientation |  | blocked - intermixed ✻ posture - identity ✻ whole figure - headless ✻ upright - inverted |  | 12.74 |  | 197.76 |  | -374.86 |  | 400.34 |  | 392 |  | 0.06 |  | .949 |  |
| group ✻ task ✻ body type ✻ gender |  | blocked - intermixed ✻ posture - identity ✻ whole figure - headless ✻ male - female |  | -158.03 |  | 197.76 |  | -545.62 |  | 229.57 |  | 392 |  | -0.80 |  | .425 |  |
| group ✻ task ✻ orientation ✻ gender |  | blocked - intermixed ✻ posture - identity ✻ upright - inverted ✻ male - female |  | 89.03 |  | 197.76 |  | -298.57 |  | 476.62 |  | 392 |  | 0.45 |  | .653 |  |
| group ✻ body type ✻ orientation ✻ gender |  | blocked - intermixed ✻ whole figure - headless ✻ upright - inverted ✻ male - female |  | 56.70 |  | 197.76 |  | -330.90 |  | 444.29 |  | 392 |  | 0.29 |  | .774 |  |
| task ✻ body type ✻ orientation ✻ gender |  | posture - identity ✻ whole figure - headless ✻ upright - inverted ✻ male - female |  | -134.90 |  | 197.76 |  | -522.50 |  | 252.69 |  | 392 |  | -0.68 |  | .496 |  |
| group ✻ task ✻ body type ✻ orientation ✻ gender |  | blocked - intermixed ✻ posture - identity ✻ whole figure - headless ✻ upright - inverted ✻ male - female |  | -124.30 |  | 395.51 |  | -899.49 |  | 650.89 |  | 392 |  | -0.31 |  | .753 |  |

Due to the significant group × gender interaction and the group × task × gender interaction (see Table A1), follow-up simple effects comparisons were performed comparing efficiency between female and male participants on the identity and posture tasks in the intermixed and blocked groups. Male participants in the blocked group were more efficient than female in the posture task (see Tables A2 and A3).

## **Table A2**

## *Follow-up Efficiency Score Comparison Between Participant Genders for Tasks in the Intermixed and Blocked Groups*

|  | |  | | | | | | 95% Confidence Interval | | | | | |  | | | | | | | | |  |
| --- | --- | --- | --- | --- | --- | --- | --- | --- | --- | --- | --- | --- | --- | --- | --- | --- | --- | --- | --- | --- | --- | --- | --- |
| Task |  | contrast | Estimate | | *SE* | | | Lower | | | Upper | | | *df* | | | *t* | | | *p* | | |  |
| Identity  intermixed  blocked | | male – female  male - female | 127.95  -47.47 |  | | 124.86  115.49 |  | | -121.28  -278.01 |  | | 377.19  183.07 |  | | 66.73  66.73 |  | | 1.02  -0.41 |  | | .309  .682 |  | |
| posture  intermixed  blocked | | male – female  male - female | 196.88  -292.76 |  | | 124.86  115.49 |  | | -52.35  -523.29 |  | | 446.12  -62.22 |  | | 66.73  66.73 |  | | 1.58  -2.53 |  | | .120  .014 |  | |
| *Note*. Orientation was kept constant | | | | | | | | | | | | | | | | | | | | | | |  |

## **Table A3**

## *Efficiency Scores for Females and Males for the Tasks in the Intermixed and Blocked Groups*

|  | | | | | | | | | | | | 95% Confidence Interval | | | |
| --- | --- | --- | --- | --- | --- | --- | --- | --- | --- | --- | --- | --- | --- | --- | --- |
| Gender | | Study | | Task | | *M* | | *SE* | | *df* | | Lower | | Upper | |
| female |  | intermixed |  | identity |  | 1101.88 |  | 66.74 |  | 66.73 |  | 968.66 |  | 1235.10 |  |
| male |  | intermixed |  | identity |  | 1229.84 |  | 105.52 |  | 66.73 |  | 1019.20 |  | 1440.48 |  |
| female |  | blocked |  | identity |  | 1150.37 |  | 72.39 |  | 66.73 |  | 1005.88 |  | 1294.87 |  |
| male |  | blocked |  | identity |  | 1102.90 |  | 89.99 |  | 66.73 |  | 923.27 |  | 1282.54 |  |
| female |  | intermixed |  | posture |  | 1693.97 |  | 66.74 |  | 66.73 |  | 1560.75 |  | 1827.19 |  |
| male |  | intermixed |  | posture |  | 1890.85 |  | 105.52 |  | 66.73 |  | 1680.21 |  | 2101.49 |  |
| female |  | blocked |  | posture |  | 1696.65 |  | 72.39 |  | 66.73 |  | 1552.15 |  | 1841.15 |  |
| male |  | blocked |  | posture |  | 1403.89 |  | 89.99 |  | 66.73 |  | 1224.26 |  | 1583.53 |  |

# **Head Interest Area Analyses with Participant Gender**

## **Table B1**

## *Head DT Proportions (Square-root Transformed): Fixed Effects Parameter Estimates for the Linear Mixed Effects Model Analysis Including Participant Gender*

|  | | | | | | | | 95% Confidence Interval | | | |  | | | | | |  |  |
| --- | --- | --- | --- | --- | --- | --- | --- | --- | --- | --- | --- | --- | --- | --- | --- | --- | --- | --- | --- |
| Names | | Effect | | Estimate | | *SE* | | Lower | | Upper | | *df* | *t* | | *p* | | |  |  |
| (Intercept) |  | (Intercept) |  | 0.46 |  | 0.02 |  | 0.41 |  | 0.51 |  | 56.12 |  | 19.79 | |  | < .001 | |  |
| group |  | blocked - intermixed |  | -0.04 |  | 0.05 |  | -0.13 |  | 0.06 |  | 56.12 |  | -0.77 | |  | .443 | |  |
| **task** |  | **posture - identity** |  | **-0.33** |  | **0.02** |  | **-0.37** |  | **-0.29** |  | **166.77** |  | **-15.92** | |  | **< .001** | |  |
| **orientation** |  | **upright - inverted** |  | **0.16** |  | **0.02** |  | **0.12** |  | **0.20** |  | **166.27** |  | **7.62** | |  | **< .001** | |  |
| gender |  | male - female |  | 0.07 |  | 0.05 |  | -0.02 |  | 0.16 |  | 56.12 |  | 1.53 | |  | .132 | |  |
| **group ✻ task** |  | **blocked - intermixed ✻ posture - identity** |  | **-0.10** |  | **0.04** |  | **-0.18** |  | **-0.02** |  | **166.77** |  | **-2.51** | |  | **.013** | |  |
| group ✻ orientation |  | blocked - intermixed ✻ upright - inverted |  | -0.03 |  | 0.04 |  | -0.11 |  | 0.05 |  | 166.27 |  | -0.62 | |  | .536 | |  |
| task ✻ orientation |  | posture - identity ✻ upright - inverted |  | 0.02 |  | 0.04 |  | -0.06 |  | 0.10 |  | 166.27 |  | 0.41 | |  | .680 | |  |
| **group ✻ gender** |  | **blocked - intermixed ✻ male - female** |  | **-0.25** |  | **0.09** |  | **-0.43** |  | **-0.07** |  | **56.12** |  | **-2.67** | |  | **.010** | |  |
| **task ✻ gender** |  | **posture - identity ✻ male - female** |  | **-0.12** |  | **0.04** |  | **-0.20** |  | **-0.04** |  | **166.77** |  | **-3.03** | |  | **.003** | |  |
| orientation ✻ gender |  | upright - inverted ✻ male - female |  | 0.02 |  | 0.04 |  | -0.06 |  | 0.10 |  | 166.27 |  | 0.59 | |  | .553 | |  |
| group ✻ task ✻ orientation |  | blocked - intermixed ✻ posture - identity ✻ upright - inverted |  | 0.00 |  | 0.08 |  | -0.16 |  | 0.16 |  | 166.27 |  | 0.01 | |  | .994 | |  |
| **group ✻ task ✻ gender** |  | **blocked - intermixed ✻ posture - identity ✻ male - female** |  | **0.26** |  | **0.08** |  | **0.10** |  | **0.42** |  | **166.77** |  | **3.16** | |  | **.002** | |  |
| group ✻ orientation ✻ gender |  | blocked - intermixed ✻ upright - inverted ✻ male - female |  | -0.05 |  | 0.08 |  | -0.21 |  | 0.11 |  | 166.27 |  | -0.58 | |  | .564 | |  |
| task ✻ orientation ✻ gender |  | posture - identity ✻ upright - inverted ✻ male - female |  | -0.02 |  | 0.08 |  | -0.18 |  | 0.14 |  | 166.27 |  | -0.24 | |  | .812 | |  |
| group ✻ task ✻ orientation ✻ gender |  | blocked - intermixed ✻ posture - identity ✻ upright - inverted ✻ male - female |  | -0.20 |  | 0.16 |  | -0.52 |  | 0.12 |  | 166.27 |  | -1.23 | |  | .219 | |  |

**For the head dwell time proportions**, the main effect of participant gender was non-significant, but due to the significant group × gender interaction and task × gender, and the group × task × gender interaction (see Table B1), follow-up simple effects comparisons were performed comparing efficiency between female and male participants on the identity and posture tasks in the intermixed and blocked groups. In the intermixed group, the male participants looked at the heads in the identity task more than the female participants (see Tables B2 and B3).

## **Table B2**

## *Follow-up Head Dwell Time Proportions (Square-root Transformed) Comparison Between Participant Genders for Tasks in the Intermixed and Blocked Groups*

|  | | |  | | | | | 95% Confidence Interval | | | |  | | | | | |  |
| --- | --- | --- | --- | --- | --- | --- | --- | --- | --- | --- | --- | --- | --- | --- | --- | --- | --- | --- |
| Task | | Gender | | Estimate | | *SE* | | Lower | | Upper | | *df* | | *t* | | *p* | |  |
| intermixed  identity  posture |  | male – female  male - female | | 0.32  0.07 |  | 0.07  0.07 |  | 0.17  -0.08 |  | 0.47  0.22 |  | 78.51  78.51 |  | 4.32  0.92 |  | < .001  .359 |  |  |
| blocked  identity  posture |  | male – female  male - female | | -0.06  -0.05 |  | 0.07  0.07 |  | -0.19  -0.19 |  | 0.08  0.09 |  | 78.51  80.07 |  | -0.81  -0.73 |  | .420  .468 |  |  |

*Note*. Orientation was kept constant.

## **Table B3**

## *Head Dwell Time Proportions (Square-root Transformed) for Females and Males for the Tasks in the Intermixed and Blocked Groups*

|  | | | | | | | | | | | | 95% Confidence Interval | | | |
| --- | --- | --- | --- | --- | --- | --- | --- | --- | --- | --- | --- | --- | --- | --- | --- |
| Gender | | Group | | Task | | *M* | | *SE* | | *df* | | Lower | | Upper | |
| female |  | intermixed |  | identity |  | 0.45 |  | 0.04 |  | 78.51 |  | 0.38 |  | 0.53 |  |
| male |  | intermixed |  | identity |  | 0.78 |  | 0.06 |  | 78.51 |  | 0.65 |  | 0.90 |  |
| female |  | blocked |  | identity |  | 0.66 |  | 0.04 |  | 78.51 |  | 0.57 |  | 0.74 |  |
| male |  | blocked |  | Identity |  | 0.60 |  | 0.05 |  | 78.51 |  | 0.50 |  | 0.71 |  |
| female |  | intermixed |  | posture |  | 0.31 |  | 0.04 |  | 78.51 |  | 0.23 |  | 0.39 |  |
| male |  | intermixed |  | posture |  | 0.38 |  | 0.06 |  | 78.51 |  | 0.25 |  | 0.50 |  |
| female |  | blocked |  | posture |  | 0.28 |  | 0.04 |  | 82.46 |  | 0.19 |  | 0.37 |  |
| male |  | blocked |  | posture |  | 0.23 |  | 0.05 |  | 78.51 |  | 0.12 |  | 0.34 |  |

# **Body Interest Area Analyses with Participant Gender**

## **Table C1**

## *Body Dwell Time Proportions (Outliers Removed): Fixed Effects Parameter Estimates for the Linear Mixed Effects Model Analysis Including Participant Gender*

|  | | | | | | | | 95% Confidence Interval | | | |  | | | | | | |  |
| --- | --- | --- | --- | --- | --- | --- | --- | --- | --- | --- | --- | --- | --- | --- | --- | --- | --- | --- | --- |
| Names | | Effect | | Estimate | | *SE* | | Lower | | Upper | | *df* | | *t* | | *p* | | |  |
| (Intercept) |  | (Intercept) |  | 0.82 |  | 0.01 |  | 0.79 |  | 0.84 |  | 56.07 |  | 66.96 |  | | < .001 |  | |
| group |  | blocked - intermixed |  | 0.01 |  | 0.02 |  | -0.03 |  | 0.06 |  | 56.07 |  | 0.54 |  | | .589 |  | |
| **task** |  | **posture - identity** |  | **0.12** |  | **0.01** |  | **0.10** |  | **0.15** |  | **390.42** |  | **8.84** |  | | **< .001** |  | |
| **body type** |  | **whole figure - headless** |  | **-0.28** |  | **0.01** |  | **-0.30** |  | **-0.25** |  | **390.42** |  | **-19.63** |  | | **< .001** |  | |
| **orientation** |  | **upright - inverted** |  | **-0.05** |  | **0.01** |  | **-0.07** |  | **-0.02** |  | **390.16** |  | **-3.35** |  | | **< .001** |  | |
| gender |  | male - female |  | -0.02 |  | 0.02 |  | -0.07 |  | 0.03 |  | 56.07 |  | -0.87 |  | | .390 |  | |
| **group ✻ task** |  | **blocked - intermixed ✻ posture - identity** |  | **0.07** |  | **0.03** |  | **0.02** |  | **0.13** |  | **390.42** |  | **2.56** |  | | **.011** |  | |
| group ✻ body type |  | blocked - intermixed ✻ whole figure - headless |  | 0.02 |  | 0.03 |  | -0.04 |  | 0.07 |  | 390.42 |  | 0.69 |  | | .491 |  | |
| **task ✻ body type** |  | **posture - identity ✻ whole figure - headless** |  | **0.37** |  | **0.03** |  | **0.32** |  | **0.43** |  | **390.42** |  | **13.36** |  | | **< .001** |  | |
| group ✻ orientation |  | blocked - intermixed ✻ upright - inverted |  | 0.03 |  | 0.03 |  | -0.03 |  | 0.08 |  | 390.16 |  | 1.07 |  | | .287 |  | |
| task ✻ orientation |  | posture - identity ✻ upright - inverted |  | 0.04 |  | 0.03 |  | -0.01 |  | 0.10 |  | 390.16 |  | 1.46 |  | | .146 |  | |
| **body type ✻ orientation** |  | **whole figure - headless ✻ upright - inverted** |  | **-0.15** |  | **0.03** |  | **-0.20** |  | **-0.09** |  | **390.16** |  | **-5.19** |  | | **< .001** |  | |
| **group ✻ gender** |  | **blocked - intermixed ✻ male - female** |  | **0.13** |  | **0.05** |  | **0.04** |  | **0.23** |  | **56.07** |  | **2.74** |  | | **.008** |  | |
| **task ✻ gender** |  | **posture - identity ✻ male - female** |  | **0.09** |  | **0.03** |  | **0.03** |  | **0.14** |  | **390.42** |  | **3.19** |  | | **.002** |  | |
| **body type ✻ gender** |  | **whole figure - headless ✻ male - female** |  | **-0.07** |  | **0.03** |  | **-0.13** |  | **-0.02** |  | **390.42** |  | **-2.64** |  | | **.009** |  | |
| orientation ✻ gender |  | upright - inverted ✻ male - female |  | -0.02 |  | 0.03 |  | -0.08 |  | 0.03 |  | 390.16 |  | -0.89 |  | | .374 |  | |
| **group ✻ task ✻ body type** |  | **blocked - intermixed ✻ posture - identity ✻ whole figure - headless** |  | **0.11** |  | **0.06** |  | **0.00** |  | **0.22** |  | **390.42** |  | **1.99** |  | | **.047** |  | |
| group ✻ task ✻ orientation |  | blocked - intermixed ✻ posture - identity ✻ upright - inverted |  | 0.00 |  | 0.06 |  | -0.10 |  | 0.11 |  | 390.16 |  | 0.09 |  | | .929 |  | |
| group ✻ body type ✻ orientation |  | blocked - intermixed ✻ whole figure - headless ✻ upright - inverted |  | 0.01 |  | 0.06 |  | -0.10 |  | 0.12 |  | 390.16 |  | 0.20 |  | | .839 |  | |
| task ✻ body type ✻ orientation |  | posture - identity ✻ whole figure - headless ✻ upright - inverted |  | 0.05 |  | 0.06 |  | -0.06 |  | 0.16 |  | 390.16 |  | 0.85 |  | | .394 |  | |
| **group ✻ task ✻ gender** |  | **blocked - intermixed ✻ posture - identity ✻ male - female** |  | **-0.18** |  | **0.06** |  | **-0.29** |  | **-0.07** |  | **390.42** |  | **-3.22** |  | | **.001** |  | |
| **group ✻ body type ✻ gender** |  | **blocked - intermixed ✻ whole figure - headless ✻ male - female** |  | **0.28** |  | **0.06** |  | **0.17** |  | **0.39** |  | **390.42** |  | **4.96** |  | | **< .001** |  | |
| **task ✻ body type ✻ gender** |  | **posture - identity ✻ whole figure - headless ✻ male - female** |  | **0.13** |  | **0.06** |  | **0.02** |  | **0.24** |  | **390.42** |  | **2.36** |  | | **.019** |  | |
| group ✻ orientation ✻ gender |  | blocked - intermixed ✻ upright - inverted ✻ male - female |  | 0.03 |  | 0.06 |  | -0.08 |  | 0.13 |  | 390.16 |  | 0.45 |  | | .654 |  | |
| task ✻ orientation ✻ gender |  | posture - identity ✻ upright - inverted ✻ male - female |  | 0.04 |  | 0.06 |  | -0.07 |  | 0.15 |  | 390.16 |  | 0.80 |  | | .425 |  | |
| body type ✻ orientation ✻ gender |  | whole figure - headless ✻ upright - inverted ✻ male - female |  | -0.04 |  | 0.06 |  | -0.15 |  | 0.07 |  | 390.16 |  | -0.76 |  | | .446 |  | |
| group ✻ task ✻ body type ✻ orientation |  | blocked - intermixed ✻ posture - identity ✻ whole figure - headless ✻ upright - inverted |  | -0.02 |  | 0.11 |  | -0.24 |  | 0.20 |  | 390.16 |  | -0.19 |  | | .852 |  | |
| **group ✻ task ✻ body type ✻ gender** |  | **blocked - intermixed ✻ posture - identity ✻ whole figure - headless ✻ male - female** |  | **-0.33** |  | **0.11** |  | **-0.55** |  | **-0.11** |  | **390.42** |  | **-2.94** |  | | **.004** |  | |
| group ✻ task ✻ orientation ✻ gender |  | blocked - intermixed ✻ posture - identity ✻ upright - inverted ✻ male - female |  | -0.02 |  | 0.11 |  | -0.24 |  | 0.20 |  | 390.16 |  | -0.18 |  | | .857 |  | |
| group ✻ body type ✻ orientation ✻ gender |  | blocked - intermixed ✻ whole figure - headless ✻ upright - inverted ✻ male - female |  | 0.07 |  | 0.11 |  | -0.15 |  | 0.29 |  | 390.16 |  | 0.64 |  | | .523 |  | |
| task ✻ body type ✻ orientation ✻ gender |  | posture - identity ✻ whole figure - headless ✻ upright - inverted ✻ male - female |  | 0.07 |  | 0.11 |  | -0.15 |  | 0.29 |  | 390.16 |  | 0.65 |  | | .515 |  | |
| group ✻ task ✻ body type ✻ orientation ✻ gender |  | blocked - intermixed ✻ posture - identity ✻ whole figure - headless ✻ upright - inverted ✻ male - female |  | 0.04 |  | 0.22 |  | -0.40 |  | 0.48 |  | 390.16 |  | 0.17 |  | | .869 |  | |

**For the body dwell time proportions**, the main effect of participant gender was non-significant. However, the group × gender interaction, task × gender interaction, body type × gender interaction, group × task × gender interaction, group × body type × gender interaction, task × body type × gender interaction, and group × task × body type × gender interaction were all significant (see Table C1). This was assessed by comparing the female and males’ body dwell time proportions for each body type in each task separately for the two groups. This revealed that for the intermixed group, compared to male participants, female participants had larger dwell time proportions to the bodies of the whole figures in the identity task. There were no significant gender differences in the blocked study (see Tables C2 to C5).

## **Table C2**

## *Follow-up Body Dwell Time Proportions Comparison Between Participant Genders for Body Types in Each Task in the Intermixed Group*

|  | | | |  | | | | | | 95% Confidence Interval | | | |  | | | | | | |  |
| --- | --- | --- | --- | --- | --- | --- | --- | --- | --- | --- | --- | --- | --- | --- | --- | --- | --- | --- | --- | --- | --- |
| Body Type | | Task | | Gender | | Estimate | | *SE* | | Lower | | Upper | | *df* | | *t* | | *p* | | |  |
| headless |  | identity |  | male - female |  | 0.00 |  | 0.04 |  | -0.08 |  | 0.09 |  | 90.70 |  | 0.07 |  | | .946 |  | |
|  |  | posture |  | male - female |  | 0.03 |  | 0.04 |  | -0.05 |  | 0.12 |  | 90.70 |  | 0.81 |  | | .422 |  | |
| whole figure |  | identity |  | male - female |  | -0.36 |  | 0.04 |  | -0.44 |  | -0.27 |  | 90.70 |  | -8.43 |  | | < .001 |  | |
|  |  | posture |  | male - female |  | -0.03 |  | 0.04 |  | -0.11 |  | 0.05 |  | 90.70 |  | -0.71 |  | | .478 |  | |
| *Note.* Orientation was kept constant. | | | | | | | | | | | | | | | | | | | | |  |

## **Table C3**

## *Body Dwell Time Proportions (Outliers Removed) for Females and Males for the Body Types in Each Task in the Intermixed Group*

|  | | | | | | | | | | | | 95% Confidence Interval | | | |
| --- | --- | --- | --- | --- | --- | --- | --- | --- | --- | --- | --- | --- | --- | --- | --- |
| Body Type | | Gender | | Task | | Mean | | *SE* | | *df* | | Lower | | Upper | |
| headless |  | female |  | identity |  | 0.98 |  | 0.02 |  | 90.70 |  | 0.94 |  | 1.03 |  |
| whole figure |  | female |  | identity |  | 0.72 |  | 0.02 |  | 90.70 |  | 0.68 |  | 0.77 |  |
| headless |  | male |  | identity |  | 0.99 |  | 0.04 |  | 90.70 |  | 0.92 |  | 1.06 |  |
| whole figure |  | male |  | identity |  | 0.36 |  | 0.04 |  | 90.70 |  | 0.29 |  | 0.43 |  |
| headless |  | female |  | posture |  | 0.90 |  | 0.02 |  | 90.70 |  | 0.85 |  | 0.94 |  |
| whole figure |  | female |  | posture |  | 0.80 |  | 0.02 |  | 90.70 |  | 0.76 |  | 0.85 |  |
| headless |  | male |  | posture |  | 0.93 |  | 0.04 |  | 90.70 |  | 0.86 |  | 1.00 |  |
| whole figure |  | male |  | posture |  | 0.77 |  | 0.04 |  | 90.70 |  | 0.70 |  | 0.85 |  |

## **Table C4**

## *Follow-up Body Dwell Time Proportions Comparison Between Participant Genders for Body Types in Each Task in the Blocked Group*

|  | | | |  | | | | | | 95% Confidence Interval | | | |  | | | | | |
| --- | --- | --- | --- | --- | --- | --- | --- | --- | --- | --- | --- | --- | --- | --- | --- | --- | --- | --- | --- |
| Task | | Body Type | | Gender | | Estimate | | *SE* | | Lower | | Upper | | *df* | | *t* | | *p* | |
| identity |  | headless |  | male - female |  | 0.01 |  | 0.05 |  | -0.10 |  | 0.11 |  | 101.10 |  | 0.10 |  | 0.920 |  |
|  |  | whole figure |  | male - female |  | 0.09 |  | 0.05 |  | -0.02 |  | 0.19 |  | 101.10 |  | 1.64 |  | 0.105 |  |
| posture |  | headless |  | male - female |  | 0.02 |  | 0.05 |  | -0.08 |  | 0.13 |  | 101.10 |  | 0.39 |  | 0.694 |  |
|  |  | whole figure |  | male - female |  | 0.07 |  | 0.05 |  | -0.04 |  | 0.18 |  | 103.41 |  | 1.30 |  | 0.195 |  |
| *Note.* Orientation was kept constant. | | | | | | | | | | | | | | | | | | | |

## **Table C5**

## *Body Dwell Time Proportions (Outliers Removed) for Females and Males for the Body Types in Each Task in the Blocked Group*

|  | | | | | | | | | | | | 95% Confidence Interval | | | |
| --- | --- | --- | --- | --- | --- | --- | --- | --- | --- | --- | --- | --- | --- | --- | --- |
| Body Type | | Gender | | Task | | *M* | | *SE* | | *df* | | Lower | | Upper | |
| headless |  | female |  | identity |  | 0.98 |  | 0.03 |  | 101.10 |  | 0.91 |  | 1.05 |  |
| whole figure |  | female |  | identity |  | 0.46 |  | 0.03 |  | 101.10 |  | 0.39 |  | 0.52 |  |
| headless |  | male |  | identity |  | 0.98 |  | 0.04 |  | 101.10 |  | 0.90 |  | 1.07 |  |
| whole figure |  | male |  | identity |  | 0.55 |  | 0.04 |  | 101.10 |  | 0.46 |  | 0.63 |  |
| headless |  | female |  | posture |  | 0.92 |  | 0.03 |  | 101.10 |  | 0.85 |  | 0.98 |  |
| whole figure |  | female |  | posture |  | 0.84 |  | 0.03 |  | 106.92 |  | 0.77 |  | 0.91 |  |
| headless |  | male |  | posture |  | 0.94 |  | 0.04 |  | 101.10 |  | 0.86 |  | 1.02 |  |
| whole figure |  | male |  | posture |  | 0.91 |  | 0.04 |  | 101.10 |  | 0.83 |  | 0.99 |  |
| *Note.* Orientation was kept constant. | | | | | | | | | | | | | | | |

# **Feet Interest Area Analyses with Gender**

## **Table D1**

## *Feet Dwell Time Proportions (Outliers Removed): Fixed Effects Parameter Estimates for the Linear Mixed Effects Model Analysis Including Participant Gender*

|  | | | | | | | | 95% Confidence Interval | | | |  | | | | | |
| --- | --- | --- | --- | --- | --- | --- | --- | --- | --- | --- | --- | --- | --- | --- | --- | --- | --- |
| Names | | Effect | | Estimate | | *SE* | | Lower | | Upper | | *df* | | *t* | | *p* | |
| (Intercept) |  | (Intercept) |  | 0.04 |  | 0.00 |  | 0.03 |  | 0.05 |  | 55.95 |  | 11.30 |  | < .001 |  |
| group |  | blocked - intermixed |  | -0.01 |  | 0.01 |  | -0.02 |  | 0.00 |  | 55.95 |  | -1.34 |  | .186 |  |
| **task** |  | **posture - identity** |  | **0.05** |  | **0.00** |  | **0.04** |  | **0.06** |  | **390.36** |  | **11.42** |  | **< .001** |  |
| **body type** |  | **whole figure - headless** |  | **-0.01** |  | **0.00** |  | **-0.02** |  | **-0.00** |  | **390.36** |  | **-2.81** |  | **.005** |  |
| **orientation** |  | **upright - inverted** |  | **-0.02** |  | **0.00** |  | **-0.03** |  | **-0.01** |  | **390.06** |  | **-3.94** |  | **< .001** |  |
| **gender** |  | **male - female** |  | **-0.02** |  | **0.01** |  | **-0.03** |  | **-0.00** |  | **55.95** |  | **-2.40** |  | **.020** |  |
| **group ✻ task** |  | **blocked - intermixed ✻ posture - identity** |  | **-0.02** |  | **0.01** |  | **-0.04** |  | **-0.00** |  | **390.36** |  | **-2.40** |  | **.017** |  |
| group ✻ body type |  | blocked - intermixed ✻ whole figure - headless |  | -0.01 |  | 0.01 |  | -0.03 |  | 0.01 |  | 390.36 |  | -1.19 |  | .237 |  |
| task ✻ body type |  | posture - identity ✻ whole figure - headless |  | -0.01 |  | 0.01 |  | -0.03 |  | 0.00 |  | 390.36 |  | -1.52 |  | .129 |  |
| group ✻ orientation |  | blocked - intermixed ✻ upright - inverted |  | -0.01 |  | 0.01 |  | -0.03 |  | 0.01 |  | 390.06 |  | -1.22 |  | .223 |  |
| **task ✻ orientation** |  | **posture - identity ✻ upright - inverted** |  | **-0.02** |  | **0.01** |  | **-0.04** |  | **-0.01** |  | **390.06** |  | **-2.59** |  | **.010** |  |
| body type ✻ orientation |  | whole figure - headless ✻ upright - inverted |  | 0.00 |  | 0.01 |  | -0.01 |  | 0.02 |  | 390.06 |  | 0.40 |  | .688 |  |
| group ✻ gender |  | blocked - intermixed ✻ male - female |  | -0.00 |  | 0.01 |  | -0.03 |  | 0.03 |  | 55.95 |  | -0.13 |  | .901 |  |
| **task ✻ gender** |  | **posture - identity ✻ male - female** |  | **-0.02** |  | **0.01** |  | **-0.04** |  | **-0.00** |  | **390.36** |  | **-2.47** |  | **.014** |  |
| body type ✻ gender |  | whole figure - headless ✻ male - female |  | 0.00 |  | 0.01 |  | -0.02 |  | 0.02 |  | 390.36 |  | 0.25 |  | .802 |  |
| orientation ✻ gender |  | upright - inverted ✻ male - female |  | 0.00 |  | 0.01 |  | -0.01 |  | 0.02 |  | 390.06 |  | 0.36 |  | .721 |  |
| group ✻ task ✻ body type |  | blocked - intermixed ✻ posture - identity ✻ whole figure - headless |  | -0.02 |  | 0.02 |  | -0.05 |  | 0.02 |  | 390.36 |  | -0.83 |  | .406 |  |
| group ✻ task ✻ orientation |  | blocked - intermixed ✻ posture - identity ✻ upright - inverted |  | -0.02 |  | 0.02 |  | -0.05 |  | 0.02 |  | 390.06 |  | -0.85 |  | .393 |  |
| group ✻ body type ✻ orientation |  | blocked - intermixed ✻ whole figure - headless ✻ upright - inverted |  | 0.01 |  | 0.02 |  | -0.02 |  | 0.05 |  | 390.06 |  | 0.71 |  | .480 |  |
| task ✻ body type ✻ orientation |  | posture - identity ✻ whole figure - headless ✻ upright - inverted |  | -0.00 |  | 0.02 |  | -0.04 |  | 0.03 |  | 390.06 |  | -0.06 |  | .952 |  |
| group ✻ task ✻ gender |  | blocked - intermixed ✻ posture - identity ✻ male - female |  | -0.00 |  | 0.02 |  | -0.04 |  | 0.04 |  | 390.36 |  | -0.01 |  | .992 |  |
| group ✻ body type ✻ gender |  | blocked - intermixed ✻ whole figure - headless ✻ male - female |  | -0.01 |  | 0.02 |  | -0.05 |  | 0.02 |  | 390.36 |  | -0.69 |  | .490 |  |
| task ✻ body type ✻ gender |  | posture - identity ✻ whole figure - headless ✻ male - female |  | -0.01 |  | 0.02 |  | -0.05 |  | 0.02 |  | 390.36 |  | -0.78 |  | .436 |  |
| group ✻ orientation ✻ gender |  | blocked - intermixed ✻ upright - inverted ✻ male - female |  | 0.04 |  | 0.02 |  | -0.00 |  | 0.07 |  | 390.06 |  | 1.92 |  | 056 |  |
| task ✻ orientation ✻ gender |  | posture - identity ✻ upright - inverted ✻ male - female |  | -0.01 |  | 0.02 |  | -0.05 |  | 0.03 |  | 390.06 |  | -0.57 |  | .570 |  |
| body type ✻ orientation ✻ gender |  | whole figure - headless ✻ upright - inverted ✻ male - female |  | -0.01 |  | 0.02 |  | -0.04 |  | 0.03 |  | 390.06 |  | -0.33 |  | .738 |  |
| group ✻ task ✻ body type ✻ orientation |  | blocked - intermixed ✻ posture - identity ✻ whole figure - headless ✻ upright - inverted |  | 0.01 |  | 0.04 |  | -0.06 |  | 0.08 |  | 390.06 |  | 0.25 |  | .802 |  |
| group ✻ task ✻ body type ✻ gender |  | blocked - intermixed ✻ posture - identity ✻ whole figure - headless ✻ male - female |  | -0.05 |  | 0.04 |  | -0.12 |  | 0.03 |  | 390.36 |  | -1.27 |  | .203 |  |
| group ✻ task ✻ orientation ✻ gender |  | blocked - intermixed ✻ posture - identity ✻ upright - inverted ✻ male - female |  | 0.07 |  | 0.04 |  | -0.00 |  | 0.14 |  | 390.06 |  | 1.92 |  | .056 |  |
| group ✻ body type ✻ orientation ✻ gender |  | blocked - intermixed ✻ whole figure - headless ✻ upright - inverted ✻ male - female |  | 0.02 |  | 0.04 |  | -0.05 |  | 0.09 |  | 390.06 |  | 0.59 |  | .555 |  |
| task ✻ body type ✻ orientation ✻ gender |  | posture - identity ✻ whole figure - headless ✻ upright - inverted ✻ male - female |  | 0.01 |  | 0.04 |  | -0.06 |  | 0.08 |  | 390.06 |  | 0.28 |  | .778 |  |
| group ✻ task ✻ body type ✻ orientation ✻ gender |  | blocked - intermixed ✻ posture - identity ✻ whole figure - headless ✻ upright - inverted ✻ male - female |  | 0.07 |  | 0.07 |  | -0.07 |  | 0.22 |  | 390.06 |  | 0.99 |  | .323 |  |

**For the feet dwell time proportions**, the main effect of participant gender was significant. Female participants looked at the feet more than the male participants. There was also a significant task × gender interaction (see Table D1). This was assessed by comparing the female and males’ feet dwell time proportions in each task. This revealed that compared to male participants, female participants had larger dwell time proportions to the feet in the posture task. There was no significant gender difference in the identity task (see Tables D2 & D3).

## **Table D2**

## *Follow-up Feet Dwell Time Proportions Comparison Between Genders in Each Task*

|  | |  | | | | | | 95% Confidence Interval | | | |  | | | | | |
| --- | --- | --- | --- | --- | --- | --- | --- | --- | --- | --- | --- | --- | --- | --- | --- | --- | --- |
| Task | | Gender | | Estimate | | *SE* | | Lower | | Upper | | *df* | | *t* | | *p* | |
| identity |  | male - female |  | -0.01 |  | 0.01 |  | -0.02 |  | 0.01 |  | 106.33 |  | -0.72 |  | .475 |  |
| posture |  | male - female |  | -0.03 |  | 0.01 |  | -0.05 |  | -0.01 |  | 106.95 |  | -3.35 |  | .001 |  |
| *Note.* Group, orientation, and body type were kept constant | | | | | | | | | | | | | | | | | |

## **Table D3**

## *Feet Dwell Time Proportions (Outliers Removed) for Females and Males in Each Task*

|  | | | | | | | | | | 95% Confidence Interval | | | |
| --- | --- | --- | --- | --- | --- | --- | --- | --- | --- | --- | --- | --- | --- |
| Gender | | Task | | *M* | | *SE* | | *df* | | Lower | | Upper | |
| female |  | identity |  | 0.02 |  | 0.00 |  | 106.33 |  | 0.01 |  | 0.03 |  |
| male |  | identity |  | 0.01 |  | 0.01 |  | 106.33 |  | -0.00 |  | 0.03 |  |
| female |  | posture |  | 0.08 |  | 0.01 |  | 108.18 |  | 0.07 |  | 0.09 |  |
| male |  | posture |  | 0.05 |  | 0.01 |  | 106.33 |  | 0.04 |  | 0.07 |  |
| *Note.* Group, orientation, and body type were kept constant | | | | | | | | | | | | | |

**References**

Bates, D., Maechler, M., Bolker, B., & Walker, S. (2015). Fitting linear mixed-effects models using lme4. *Journal of Statistical Software, 67*(1), 1-48. doi: 10.18637/jss.v067.i01

jamovi module. (2019). *GAMLj: General Analyses for Linear Models.* Available: <https://gamlj.github.io/>

R Core Team. (2019). *R: A Language and Environment for Statistical Computing*. Available: <https://cran.r-project.org/>.
